# Cardiometabolic Risk in Chronic Spinal Cord Injury: A Systematic Review with Meta-Analysis and Temporal and Geographical Trends

**DOI:** 10.3390/jcm14092872

**Published:** 2025-04-22

**Authors:** Gary J. Farkas, Lizeth J. Caldera, Daniel D. Hodgkiss, Jessica R. Mitchell, Thomas F. Pelaez, Maxwell A. Cusnier, Alex J. Cole, Scott G. Daniel, Matthew T. Farrow, Cameron M. Gee, Eric A. Kincaid-Sharp, Andrew Malcolm Green Logan, David W. McMillan, Tom E. Nightingale, Brieanna Perdue, Pauline Portes, Francis T. Walson, Alyssa M. Volmrich, John M. Reynolds, Mark S. Nash, David R. Gater, Arthur S. Berg

**Affiliations:** 1 Department of Physical Medicine and Rehabilitation, Leonard M. Miller School of Medicine, University of Miami, Miami, FL 33136, USA; 2 The Miami Project to Cure Paralysis, Department of Neurological Surgery, Leonard M. Miller School of Medicine, University of Miami, Miami, FL 33136, USA; 3 School of Sport, Exercise, and Rehabilitation Sciences, University of Birmingham, Edgbaston, Birmingham B15 2TT, UK; 4 School of Osteopathic Medicine, Rowan-Virtua University, Stratford, NJ 08084, USA; 5 DeBusk College of Osteopathic Medicine, Lincoln Memorial University, Knoxville, TN 37752, USA; 6 Leonard M. Miller School of Medicine, University of Miami, Miami, FL 33136, USA; 7 Department of Physical Medicine and Rehabilitation, The Ohio State University, Columbus, OH 43210, USA; 8 Department of Orthopaedics, Faculty of Medicine, University of British Columbia, Vancouver, BC V6T 1Z4, Canada; 9 Centre for Trauma Science Research, University of Birmingham, Edgbaston, Birmingham B15 2TT, UK; 10 Jerry M. Wallace School of Osteopathic Medicine, Campbell University, Lillington, NC 27546, USA; 11 Department of Physical Medicine and Rehabilitation, School of Medicine, MetroHealth Rehabilitation Institute, Case Western Reserve University, Cleveland, OH 44106, USA; 12 Louis Calder Memorial Library, Leonard M. Miller School of Medicine, University of Miami, Miami, FL 33136, USA; 13 Department of Public Health Sciences, College of Medicine, The Pennsylvania State University, Hershey, PA 17033, USA

**Keywords:** spinal cord injury, tetraplegia, paraplegia, cardiometabolic risk, cardiometabolic syndrome, risk factors, obesity, insulin resistance, blood pressure, cholesterol, lipids, cardiovascular disease, glucose metabolism, athletes

## Abstract

**Objectives**: This systematic review with meta-analysis compared cardiometabolic syndrome (CMS) in adults with chronic (≥1 year) spinal cord injury (SCI) to non-SCI individuals (controls) and athletes, analyzing the effect of specific injury characteristics and exploring temporal and geographical trends. **Methods**: Ovid Medline, Embase, Cochrane, CINAHL, Scopus, and Web of Science were searched from inception to September 2024. Adults with chronic SCI were included based on observational and baseline data derived from experimental studies. Quality Assessment Criteria for Evaluating Primary Research Papers from a Variety of Fields assessed quality. Weighted means with 95% bootstrapped confidence intervals (CI) were computed for risk stratification. Group differences were assessed using random effects meta-analysis, calculating weighted mean differences with 95% bootstrapped CI. Temporal and geographical trends were evaluated with linear regression based on sample-size-weighted distributions and relevant covariates. **Results**: Of 31,163 identified records, 471 studies were included (*n* ≤ 31,782 SCI participants). CMS was present in men with SCI, paraplegia, tetraplegia, and injuries above T6; men with complete SCI (AIS A); and men and women with motor-complete SCI (AIS A–B). Compared to controls, adults with SCI had a lower body mass index (BMI), higher total and visceral fat, and worse lipid and carbohydrate profiles, including increased insulin resistance (IR). Tetraplegia was associated with greater visceral fat, poorer glycemic control, and lower BMI, insulin sensitivity, high-density lipoprotein-cholesterol (HDL-C), and triglycerides than paraplegia. Motor-complete SCI had lower BMI, HDL-C, and fasting glucose than motor-incomplete injuries. Injuries above T6 had lower blood pressure and higher fasting insulin levels than those below T6. Athletes with SCI had a lower BMI, fat mass, and fasting glucose, and higher systolic blood pressure than non-athletes with SCI, but frequently presented with obesity and carbohydrate dysfunction. Temporal analysis revealed increasing obesity trends and improved systolic blood pressure, while other CMS risk factors remained unchanged. We also identified global variations in obesity, lipids, blood pressure, and carbohydrate patterns. **Conclusions**: With a large sample, we revealed a widespread cardiometabolic burden in chronic SCI, even among athletes. Specifically, obesity, IR, and hypoalphalipoproteinemia worsened with increasing injury severity, alongside rising obesity trends and geographic disparities in risk profiles. These patterns highlight the evolution of what was deemed an epidemic into a global cardiometabolic pandemic.

## 1. Introduction

A spinal cord injury (SCI) disrupts motor, sensory, and autonomic pathways, leading to paralysis and accelerated aging. Post-injury immobility, autonomic dysfunction [1], sublesional myopenia [2], sedentary behavior [3], and overeating [4] relative to reduced energy expenditure [5,6,7] contribute to neurogenic obesity [8,9]. This obesity phenotype is a major contributor to cardiometabolic risk in SCI [9,10,11], including disturbances in lipid metabolism, glucose regulation, and vascular health. When three or more cardiometabolic disorders converge, cardiometabolic syndrome (CMS) emerges, characterized by obesity, hypertriglyceridemia, hypoalphalipoproteinemia, hypertension, and insulin-resistant glucose metabolism/dysglycemia [10,11,12]. Among those with SCI, CMS significantly increases the risk of cardiovascular disease over time [12,13]. Notably, 80% of CMS-related risk factors progress silently, without overt symptoms [14,15], highlighting the persistent and often subclinical cardiovascular burden associated with SCI.

Understanding cardiovascular disease progression requires examining temporal trends and geographic variations in cardiometabolic risk to track subclinical and clinical stages. To the authors’ knowledge, such research is lacking in SCI. Recruiting individuals with SCI for clinical trials is challenging due to persistent health conditions and secondary complications that affect independence and participation, while limited catchment areas and transportation barriers further restrict enrollment [16,17,18,19]. These factors reduce sample sizes, limit longitudinal follow-up, and hinder the ability to generalize geographic risk comparisons, resulting in predominantly cross-sectional CMS studies [8,20,21,22,23,24,25,26,27,28,29,30,31,32,33,34,35]. Injury heterogeneity, including neurological and autonomic factors, also complicates generalizability across SCI subpopulations, including athletes. Despite inherent limitations, meta-analyses enhance statistical power by aggregating data across studies [36], offering broader insights to inform evidence-based care.

While systematic reviews and meta-analyses have examined health risks in individuals with SCI [37,38,39,40,41], they often focus on statistical differences in risk profiles compared to non-SCI populations. Many overlook a critical factor: the specific diagnostic thresholds for CMS components [11], which are more clinically relevant in assessing cardiovascular disease risk. These risks likely drive global disparities in cardiovascular morbidity and mortality during chronic SCI, yet the extent of these differences remains insufficiently characterized [42,43,44,45,46,47,48,49,50,51,52,53,54,55,56]. Here, we assess these variations within the CMS framework, analyzing group-level differences and risk factor trends over time in adults with chronic SCI. This approach quantifies the extent of cardiometabolic risk in chronic SCI, highlighting disparities that delineate patterns to guide targeted prevention and management strategies.

This systematic review with meta-analysis aimed to assess cardioendocrine health components in adults with chronic SCI, specifically emphasizing cardiometabolic risk and CMS. This study had three specific objectives towards this aim. The first objective was to assess the burden of cardiometabolic risk, focusing on the component risk factors for CMS in adults with chronic SCI and stratified based on neurological and sympathetic injury levels (above or below T6), injury severity, and identification as an athlete. The second objective explored temporal and geographical trends in the component risk factors for CMS across the general, non-athletic population with chronic SCI. The third objective was to compare the same cardiometabolic parameters from objective one between individuals with and without (“controls”) chronic SCI, the above-referenced groups, and athletes and the general, non-athletic population with chronic SCI. We hypothesized that cardiometabolic risk would worsen with increasing injury severity, with greater risk observed in individuals with complete injuries, higher injury levels, and non-athletic status, as evidenced by obesity, vascular health, lipid profiles, and carbohydrate metabolism.

## 2. Methods

This systematic review with meta-analysis was specified *a priori* via pre-registration on PROSPERO (CRD42022365344). It was conducted with guidance from the Methodological Expectations of Cochrane Intervention Reviews [57] and reported following the Preferred Reporting Items for Systematic Review and Meta-Analyses (PRISMA) [58]. The PRISMA checklist is presented in the Supplementary Materials.

### 2.1. Study Outcomes

Table 1 presents the predefined primary outcomes. Secondary outcomes (body mass index [BMI], body mass, and height) were analyzed only when studies reported at least one primary outcome.

### 2.2. Study Eligibility Criteria

Studies included adults (≥18 years) with traumatic or non-traumatic chronic SCI, defined as a minimum duration of 12 months post-injury [4]. To ensure a comprehensive search strategy and a robust pool of studies and participants, we included experimental (interventional, randomized controlled, crossover trials) and observational (cohort, cross-sectional) studies. Studies were excluded if they were (1) a case report (*n* = 1) or case series (*n* ≤ 5) because of a low level of evidence and a higher potential for bias [59]; (2) published conference proceedings; (3) heterogeneous study samples including non-SCI neurological conditions (e.g., multiple sclerosis) or acute SCI (<12 months post-injury), unless data were presented separately; (4) review articles (narrative, scoping, meta-analyses, systematic reviews); (5) duplicate studies; and (6) commentaries. Additionally, studies were excluded if they involved non-human participants, participants under 18 years, or did not report specific cardiometabolic outcomes. Studies with incomplete data, such as reporting means without standard deviations or missing data for summary statistics, were also excluded. Non-English studies without available translations were also excluded.

### 2.3. Study Searches

The search strategy was developed by an academic health science librarian (JMR) in consultation with the corresponding author (GJF). It was reviewed by an independent medical librarian using the Peer Review for Electronic Search Strategies tool [60]. A preliminary search of electronic databases was conducted to refine the keywords and enhance the sensitivity of terms used in the search strategy. The primary search strategy was written for Ovid Medline and translated using each database’s syntax, controlled vocabulary, and search fields. Medical Subject Headings terms, EMTREE terms, and text words were used for the concepts of spinal cord injuries, tetraplegia, paraplegia, a wide variety of obesity, diabetes, and metabolic measurements, and their synonyms. We searched Ovid Medline (Medline Epub-Ahead-of-Print, In-Process and Other Non-Indexed Citations and Daily), Embase (Elsevier, Embase.com), Cochrane Central Register of Controlled Trials (CENTRAL; Cochrane Library, Wiley, all issues), CINAHL Plus with Full Text (Ebsco), Scopus (Elsevier), and the Web of Science platform. The Medline search strategy was adapted for other databases, in part, with the use of the Institute for Evidence-Based Health Care’s Polyglot Search translator [61]. No language, date, or other limits were applied at the search phase. We filtered for English-language studies during screening. All databases were searched from their inception until 27 September 2024. All database records were downloaded to EndNote 21 [62] and uploaded to Covidence Web-based software (www.covidence.org, accessed on 27 July 2021) [63] to remove duplicates and for screening, full-text evaluation, data extraction, and quality assessment. Due to the large number of studies and limited past success in retrieving missing data [64,65], we did not contact the corresponding authors when information was incomplete (see details below). The Retraction Watch database (retractiondatabase.org) was queried with EndNote software (versions 20 and 21) for retractions of included studies. We did not contact manufacturers, other experts, or search study registries. The full search strategy is provided as Supporting Information (File S1).

### 2.4. Study Selection

After removing duplicates and piloting the title and abstract screening procedure to ensure consistency across reviewers, reviewers independently assessed studies for inclusion using Covidence. Each study was assessed by title and abstract independently by 2 randomly paired reviewers out of the 15 reviewers (JRM, TFP, MAC, AJC, SGD, ZJD, MTF, DDH, BP, CMG, EAKS, AMGL, PP, FSW, and AV). Studies with unclear eligibility during the title and abstract screening were retained for full-text screening. Two randomly paired reviewers from a pool of 13 (LJC, JRM, TFP, MAC, AJC, ZJD, MTF, DDH, BP, EAKS, PP, FSW, and AV) conducted the full-text review of included studies. A third reviewer (GJF) resolved any conflicts regarding study inclusion/exclusion. Full inaccessible texts were obtained through database searches via Interlibrary Loan services. The full-text requests were submitted by two authors (GJF or MAC) and were fulfilled by the University of Miami Louis Calder Memorial Library and the Florida State University Library System.

### 2.5. Data Extraction and Management

A data extraction template in Covidence was used to extract data from eligible studies in duplicate by 2 randomly paired reviewers from a pool of 14 independent reviewers (LJC, JRM, TFP, ZJD, MTF, DDH, CMG, EAKS, BP, CMM, EP, PP, FSW, and AV). After extraction, a single reviewer (LJC, JRM, or TFP) not involved in the original process cross-checked the data using Covidence’s consensus feature. The same reviewer resolved discrepancies or, if needed, discussed them with the lead author (GJF). Only baseline data were extracted from studies with a longitudinal design. We reviewed author names and study methods with overlapping participants to identify redundancies, prioritizing the most recent or most relevant publication for data extraction [66].

Extracted data reported as a five-number summary (median, quartiles, minimum, and maximum) were converted to means and standard deviations using a standardized method accounting for sample sizes [67,68,69,70]. A single reviewer (LJC, JRM, or TFP) validated the accuracy of these conversions during the consensus process within Covidence. Extracted items included primary and secondary outcomes, study details (title, first author, year, study design, journal, geographical region), aim/objective, sample size, demographics (age, sex), injury characteristics (time since injury, injury level, injury severity), assessment methods for total body fat percentage (TBFP) and insulin sensitivity, and data for each individual subgroup, where possible. These subgroups were as follows: (1) neurological level of injury (tetraplegia vs. paraplegia); (2) injury completeness (sensory/motor-complete vs. sensory/motor incomplete SCI); (3) motor-completeness (motor-complete vs. motor-incomplete SCI); (4) sympathetic level of injury (above and below T6); and (5) athletic status (athletes with chronic SCI vs. the general, non-athletic population with chronic SCI). We classified the neurological level of injury according to the International Standards for Neurological Classification of Spinal Cord Injury (ISNCSCI) [71]. Specifically, persons with tetraplegia were identified as having cervical injuries from C1 to C8, while paraplegia encompassed all thoracic, lumbar, and sacral segments of the spinal cord [71]. Motor and sensory complete injuries (American Spinal Injury Association Impairment Scale (AIS) A) were distinguished from incomplete injuries (AIS B, C, and D) based on the AIS. Additionally, motor-complete injuries (AIS A and B) were differentiated from motor-incomplete injuries (AIS C and D). While variability exists, we classified sympathetic injury levels “functionally”, defining injuries at or above T6 as high and those below as low [72]. T6 represents the upper limit of splanchnic sympathetic outflow and adrenal innervation, critical for vascular tone and metabolism. Injuries at or above this level disrupt catecholamine release [73,74], whereas lower injuries preserve adrenal and visceral sympathetic function. Neuroanatomy and empirical evidence justify T6 as a key threshold for classifying sympathetic impairment in SCI. This cutoff aligns with cardiac sympathetic efferents (T1–T5) and is supported by its association with autonomic dysreflexia, dyslipidemia, insulin resistance, and reduced energy expenditure [75]. Athlete data were excluded from general chronic SCI analyses, as they do not reflect the typically inactive SCI population [76,77,78]. Studies including mixed SCI cohorts (e.g., tetraplegia and paraplegia, complete and incomplete, motor-complete and motor-incomplete) were categorized as ‘SCI’ or ‘not reported/cannot determine’ when subgroup differentiation was not possible. Studies with this classification were not included in the subgroup analyses but were included in the analysis comparing adults with chronic SCI to controls without SCI. Controls without SCI and their associated data were extracted from studies when such participants were present in the studies that met the inclusion criteria for this paper.

### 2.6. Quality Assessment

Quality/bias assessment for each study was evaluated based on the study design, data management, and reporting bias using the Standard Quality Assessment Criteria for Evaluating Primary Research Papers from a Variety of Fields [79]. This tool was selected because of its standard reproducible criteria for critically appraising a wide range of studies [79]. The instrument comprised 14 items evaluated on a 3-point scale (2 = Yes, 1 = Partial, 0 = No) to assess the fulfillment of each criterion, with considerations in place for when some items were marked as not applicable. A summary score was calculated for each study by summing the total score obtained across relevant items, dividing by the total possible score (i.e., 28 − [number of n/a × 2]), and multiplying by 100. We categorized studies into 10 clusters, employing a 0% and 100% scale, representing the lowest and highest quality ratings, respectively. Two out of seven reviewers completed all quality/bias assessments in Covidence in duplicate (LMC, JRM, TFP, MAC, BP, MTF, and DDH). All disagreements/conflicts were resolved via a group discussion among the reviewers.

### 2.7. Cardiometabolic Risk Stratification in SCI

CMS risk stratification was conducted based on the pooled data following the Consortium for Spinal Cord Medicine Clinical Practice Guidelines on the Identification and Management of Cardiometabolic Risk after SCI [11] and pertinent medical criteria for risk identification at standard age and sex risk strata. Consistent with established authoritative standards [11], CMS was defined as the presence of three or more of the following: obesity, dysglycemia or insulin resistance, dyslipidemia, and hypertension. We operationalized obesity as a TBFP > 22% in men and >35% in women [11], an SCI-specific BMI > 22 kg/m^2^ [11,80], or visceral fat (>1630 mL) [23]. We characterized dyslipidemia as the presence of hypertriglyceridemia with triglycerides ≥ 150 mg/dL or hypoalphalipoproteinemia with HDL-C < 40 mg/dL for men or <50 mg/dL for women [11,12]. Hypertension was identified as systolic or diastolic blood pressure ≥ 130 or ≥85 mmHg, respectively [11,12]. Dysglycemia was defined as fasting blood glucose > 100 mg/dL [11], hemoglobin A1c (HbA1c) in the range of 5.7–6.4% (prediabetic) or ≥6.5% (type 2 diabetes mellitus), or an oral glucose tolerance test (OGTT) result ranging between 140 and 199 mg/dL (prediabetes) or >200 mg/dL (type 2 diabetes mellitus) [81]. Insulin resistance was operationalized utilizing established indices [82,83], including HOMA1 (1.7–2.4 for hepatic and >2.5 for significant insulin resistance), HOMA2 (insulin resistance ≥ 1.4), quantitative insulin sensitivity check index (QUICKI; insulin resistance < 0.339), and Matsuda index (insulin resistance ≤ 2.5). We compared weighted averages against established CMS diagnostic thresholds to contextualize our findings clinically.

### 2.8. Statistical Analysis

Data were exported from Covidence into Excel for Microsoft 365 (version 2307, Microsoft Corp., Redmond, WA, USA) and imported into R (version 4.3.2, R Core Team, Vienna, Austria, 2022) [84]. Before initiating the formal analysis, a series of data validation and quality assurance queries were executed in R. These queries encompassed data checks and confirmation procedures to identify and rectify errors or discrepancies within the dataset. If an error was detected, we cross-referenced and verified the data directly from the original study and updated the database as needed.

For Aim 1, we report weighted means (weighted by study sample size) and 95% bootstrapped confidence intervals for all outcome metrics calculated according to previously published methods [64]. We compared weighted averages against CMS diagnostic thresholds at the population level for each subgroup (SCI, tetraplegia, paraplegia, complete, incomplete, motor-complete, motor-incomplete, above and below T6, athletes) and stratified by sex. These weighted values, derived from observational and baseline data in longitudinal studies, were independent of the meta-analysis calculations to leverage a larger data pool for more refined risk stratification.

For Aim 2, we used ggplot2 (v3.4.4) [85] in R to generate graphics illustrating the temporal and geographical distributions of CMS risk factors, weighted by the respective sample size of each study. Linear regression models, weighted by sample size and adjusted for age, weight, the proportion of men, and the proportion of tetraplegia, were used to analyze temporal trends, with the trend *p*-value reported. To optimize the sample size to examine geographical variation in cardiometabolic risk, we categorized regions into Asia (India, Japan, South Korea, Taiwan, Thailand), Australasia (Australia and New Zealand), Europe (Austria, Denmark, Italy, Germany, Greece, Netherlands, Poland, Spain, Sweden, United Kingdom), and the Middle East (Iran, Israel, Turkey). Brazil, Canada, and the United States were analyzed separately due to their large sample sizes, with data aggregated from multiple studies. South Africa was excluded from the geographical analysis due to a limited number of studies (*n* = 2). Geographical variation in cardiometabolic risk was analyzed using linear regression models, weighted by sample size and adjusted for publication year, age, weight, proportion of men, and tetraplegia proportion. Given its sample size, the US was chosen as the reference SCI population, providing a robust basis for comparison. We report the beta coefficients (β) with standard errors (SE) and *p*-values. Positive β values indicate higher levels of the cardiometabolic risk factor in the specified region compared to the US. In contrast, negative β values indicate lower levels than in the US.

For the quantitative analysis in Aim 3, a random-effects meta-analysis was conducted using the rma function from the metafor package [86] with the DerSimonian and Laird method [87] to estimate the overall effect size for each variable across all studies. This meta-analysis included observational and baseline longitudinal data, requiring at least two studies with a suitable comparison group [88,89]. Summary measures include total sample size per comparison group, study count, weighted mean difference (WMD) with 95% bootstrapped confidence intervals, and weighted mean and standard deviation (derived from relevant studies in each meta-analysis comparison). A positive WMD indicates higher aggregated means in individuals with more severe health status (e.g., tetraplegia, motor-complete SCI) than those with less severe status (e.g., paraplegia, motor-incomplete SCI). In contrast, a negative WMD indicates the opposite. Heterogeneity was evaluated using the I^2^ statistic and categorized based on the Cochrane Collaboration guideline as low (I^2^ = 0–40%), moderate (I^2^ = 30–60%), substantial (I^2^ = 50–90%), and considerable heterogeneity (I^2^ = 75–100%) [90]. The significance level was set at an α ≤ 5% for all analyses. All statistical analyses were conducted using R [84].

## 3. Results

### 3.1. Search Results

**Figure 1:**
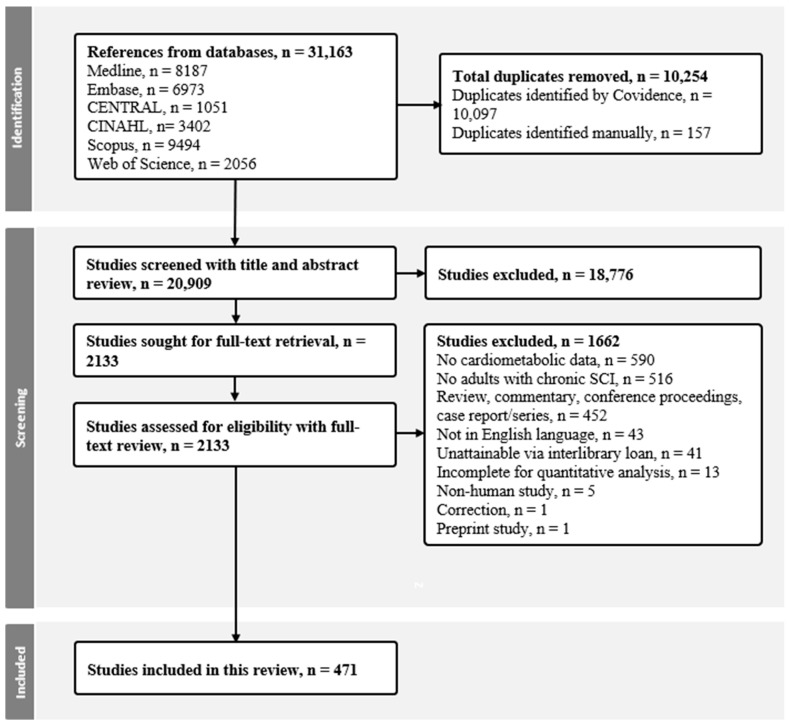
PRISMA flow diagram. Preferred Reporting Items for Systematic Review and Meta-Analyses (PRISMA) flow diagram.

Figure 1 presents the PRISMA flow chart for study inclusion. Of 31,163 studies identified across six databases, 20,909 remained after duplicate removal. Following title and abstract screening, 18,776 were excluded, leaving 2133 for full-text review. Among these, 1662 were excluded after a full-text assessment based on the eligibility criteria, resulting in the inclusion of 471 studies that met all eligibility criteria for analysis. A full list is available in Supporting Information (File S2).

### 3.2. Study Descriptions

**Figure 2:**
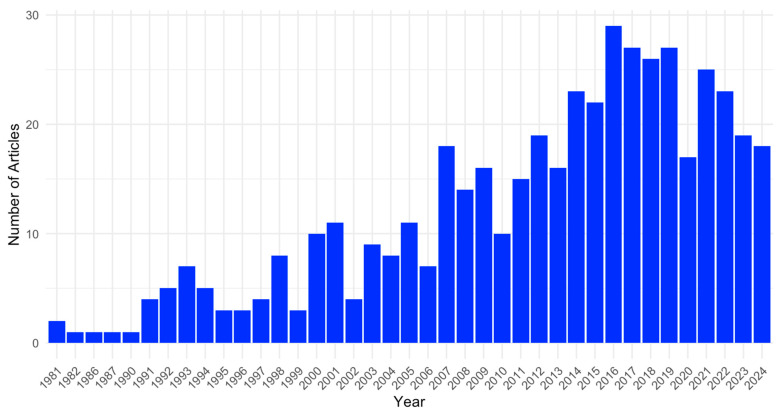
Publication timeline. The temporal series of published studies of this systematic review with meta-analysis. The x-axis represents the time in years from the inception of the six searched databases to September 2024. The y-axis depicts the cumulative number of included articles for a given year.

Figure 2 illustrates the annual distribution of studies from 1981 to 2024 to contextualize the scope of included evidence. The peak publication year was 2016, with a median of 2014. The first and third quartiles were 2007 and 2019, respectively, and the mean publication year was 2012.

Concerning study design, this review included 322 (68.4%) cross-sectional studies, 40 (8.5%) cohort studies, 54 (11.5%) pre-post studies, 34 (7.2%) RCTs, 20 (4.2%) crossover studies, and 1 (0.2%) non-RCT. While sample sizes varied based on each weighted analysis, the evidence base was most extensive for weighted age (*n* ≤ 31,782), time since injury (*n* ≤ 28,477), and BMI (*n* ≤ 28,295), and smallest for percent (*n* ≤ 64) and absolute (*n* ≤ 42) intramuscular adipose tissue. Regarding sex, 88.2% of the sample were adult males. The weighted mean age was 48.9 years (44.6–52.5) and 16.8 years (14.8–18.2) for time post-SCI.

Of the 471 studies, 190 (40.3%) investigated at least one body composition outcome (excluding body mass, height, and BMI), whereas body mass, height, and BMI were assessed in 314 (66.7%), 262 (55.6%), and 295 (62.6%) studies, respectively. Overall, 215 (45.7%) provided data on at least one vascular outcome, 162 (34.4%) reported at least one lipid marker, 138 (29.3%) examined at least one glycemic parameter, and 61 (13.0%) included at least one variable of inflammation (File S2).

Across 155 studies, various techniques were employed to assess TBFP. Dual-energy X-ray absorptiometry (DXA) was featured in 120 (77.4%) studies, bioelectrical impedance was applied in 21 (13.5%) studies, skinfold technique was referenced in 6 (3.9%) studies, hydrometry (isotope dilution) was used in 4 (2.6%) studies, and air displacement plethysmography was used in 2 (1.3%) studies. Additionally, one study utilized a prediction equation (0.6%), and another used underwater/hydrostatic weighing (0.6%). Visceral fat was assessed in 27 studies with ultrasound (*n* = 1, 3.7%), DXA (*n* = 13, 48.1%), computed tomography (*n* = 2, 7.4%), and magnetic resonance imaging (*n* = 11, 40.7%). Thirty-one studies assessed advanced glucose-insulin dynamics using hyperinsulinemic-euglycemic clamp (*n* = 1, 3.2%), intravenous glucose tolerance test (*n* = 6, 19.4%), and OGTT (*n* = 24, 77.4%) (File S2).

### 3.3. Quality Assessment Results

The mean quality rating was 90% (62–100%), with an SD of 10%. Two hundred and eighty-six studies, comprising 60.6% of the total, achieved a score between 90 and 100%. Meanwhile, 141 (29.9%) studies scored between 80 and 90%, and 54 (11.4%) were between 70 and 80%. Lastly, the quality of 24 (5.1%) studies was rated between 60 and 70%. Full ratings for each study can be found in Supporting Information (File S3).

### 3.4. Weighted Risk Factors of Cardiometabolic Syndrome (Aim 1)

This section presents Aim 1 findings on weighted CMS risk factors and aggregate CMS diagnosis across subgroups (Table 2). The Supporting Information (Files S4–S9) provides detailed weighted averages, sample sizes, and references for cardiometabolic health measures in the aggregated general, non-athletic chronic SCI population, categorized by subgroup and sex.

General, Non-Athletic Population with Chronic SCI: In the general, non-athletic population with chronic SCI, BMI was 25.5 kg/m^2^ (25.1–25.7 kg/m^2^; *n* = 25,295), visceral fat measured 1465.5 mL (1214.5–1675.2 mL; *n* = 1289), and TBFP was 31.1% (28.9–32.8%; *n* = 2130) in men and 36.9% (35.1–45.6%; *n* = 107) in women. Systolic and diastolic blood pressure demonstrated weighted averages of 122.4 mmHg (115.9–125.0 mmHg; *n* = 15,503) and 72.3 mmHg (70.7–73.6; *n* = 15,584), respectively. Triglycerides were 125.9 mg/dL (121.4–130.0 mg/dL; *n* = 10,878), while HDL-C levels were 39.6 mg/dL (38.8–40.5 mg/dL; *n* = 3746) in men and 52.7 mg/dL (50.9–55.2 mg/dL; *n* = 372) in women. Further, fasting glucose and HbA1c were measured at 95.5 mg/dL (93.2–98.6 mg/dL; *n* = 6793) and 5.5% (5.4–5.6%; *n* = 1914), respectively. Regarding insulin resistance, the Matsuda Index was 4.7 (3.7–5.8; *n* = 137), HOMA1 was 2.2 (1.9–2.5; *n* = 1178), and HOMA2 was 1.4 (1.2–1.6; *n* = 185). Using QUICKI, insulin sensitivity was 0.7 (0.4–1.5; *n* = 119). Regarding CMS risk stratification, weighted values for obesity exceeded BMI and body fat cutoffs in both men and women, classifying the pooled sample as obese (Table 2). Men met the criteria for low HDL-C, and HOMA1 and HOMA2 indicated insulin resistance. Overall, the general, non-athletic chronic SCI population met CMS criteria for obesity, insulin resistance, and low HDL-C in men.

Paraplegia: Persons with paraplegia exhibited obesity, with a BMI of 24.9 kg/m^2^ (24.6–25.3 kg/m^2^; *n* = 3927), TBFP of 27.9% (25.9–30.2%; *n* = 405) in men and 45.4% (45.3–45.5%; *n* = 12) in women, and visceral fat of 1295.9 mL (782.2–1752.2 mL, *n* = 221). Systolic blood pressure was 118.4 mmHg (110.6–124.8 mmHg; *n* = 1694), while diastolic blood pressure was 71.7 mmHg (64.0–76.1; *n* = 1675). Triglycerides were 128.6 mg/dL (122.5–134.7 mg/dL; *n* = 2331), and HDL-C was 38.4 mg/dL (37.0–40.1 mg/dL; *n* = 970) and 60.0 mg/dL (59.9–60.0 mg/dL; *n* = 48) in men and women, respectively. Fasting glucose levels were 94.1 mg/dL (91.7–96.9 mg/dL; *n* = 1565), and HbA1c was 5.6% (5.4–5.7%; *n* = 343). HOMA1 yielded a 2.1 (1.7–2.7; *n* = 393), while the HOMA2 was calculated as 1.1 (0.9–1.2; *n* = 41). In summary, men and women with paraplegia met CMS criteria for obesity (BMI and TBFP) and HOMA1 insulin resistance, while only men met the criteria for hypoalphalipoproteinemia. Thus, men with paraplegia meet the criteria for CMS (Table 2).

Tetraplegia: In individuals with tetraplegia, BMI was 24.7 kg/m^2^ (24.0–25.3 kg/m^2^; *n* = 2402), and TBFP was 26.8% (24.0–30.5%; *n* = 176) in men (no data were available for women with tetraplegia), and 1774.0 mL (1321.4–2463.0 mL, *n* = 97) for visceral fat. Systolic and diastolic blood pressure had weighted averages of 110.6 mmHg (106.1–113.7 mmHg; *n* = 939) and 67.7 mmHg (65.2–69.4; *n* = 919), respectively. Triglyceride levels were 129.5 mg/dL (121.3–136.1 mg/dL; *n* = 1465), and HDL-C was 39.1 mg/dL (36.1–41.0; *n* = 368) in men and 58.9 mg/dL (58.7–59.0; *n* = 16) in women. Fasting glucose and HbA1c were 97.1 mg/dL (91.9–103.0 mg/dL; *n* = 607) and 5.5% (5.3–5.7%; *n* = 205), respectively. Insulin resistance quantified through the HOMA1 was 1.8 (1.3–2.3 mg/dL; *n* = 105). In individuals with tetraplegia, BMI, TBFP, and visceral fat exceeded obesity thresholds. Men, but not women, had HDL-C levels indicative of hypoalphalipoproteinemia. Thus, men with tetraplegia met CMS criteria for obesity, insulin resistance, and hypoalphalipoproteinemia (Table 2).

Incomplete Injuries: Examining individuals with incomplete injuries, BMI was 25.3 kg/m^2^ (24.1–26.2 kg/m^2^; *n* = 313), whereas an aggregated TBFP for both men and women was 31.0% (25.3–35.0%; *n* = 79) (no sex-specific data were available for persons with incomplete SCI). Systolic and diastolic blood pressure averaged 113.2 mmHg (110.7–116.9 mmHg; *n* = 44) and 69.9 mmHg (67.2–72.6; *n* = 44), respectively. Triglycerides were 122.6 mg/dL (108.9–127.6 mg/dL; *n* = 224), and HDL-C in men was 45.4 mg/dL (*n* = 23) (insufficient data were available for women). Fasting glucose was 97.8 mg/dL (88.7–103.2 mg/dL; *n* = 109), and HOMA1 was 2.6 (1.5–4.6; *n* = 35). Individuals with incomplete SCI met CMS criteria for obesity (BMI and TBFP) and insulin resistance (HOMA1) (Table 2).

Complete Injuries: BMI was 24.0 kg/m^2^ (23.3–24.5 kg/m^2^; *n* = 1030) with a TBFP of 27.9% (23.7–32.9%; *n* = 100) in men (no data were available for women). Systolic and diastolic blood pressure were quantified at 117.6 mmHg (111.7–122.0 mmHg; *n* = 340) and 71.9 mmHg (69.4–73.7; *n* = 331), respectively. Triglycerides were 123.1 mg/dL (118.9–130.0 mg/dL; *n* = 631), while HDL-C was calculated at 36.0 mg/dL (33.1–39.6 mg/dL; *n* = 194) in men (no data were available for women). Fasting glucose levels were 90.2 mg/dL (84.5–94.1 mg/dL; *n* = 350), and HOMA1 was 2.1 (1.4–3.4; *n* = 156). Men with complete SCI met CMS criteria for obesity (BMI and TBFP), insulin resistance (HOMA1), and low HDL-C based on aggregated values (Table 2).

Motor-incomplete Injuries: In individuals with motor-incomplete SCI, the BMI measured 25.9 kg/m^2^ (23.4–26.7; *n* = 141), and one study reported TBFP of 31% (*n* = 36) (no data were available by sex). Systolic blood pressure was 118 mmHg, and diastolic blood pressure was 66 mmHg, according to one study (*n* = 6). Triglyceride levels were 127.3 mg/dL (100.8–133.8 mg/dL; *n* = 105), and HDL-C levels in men were 47.8 mg/dL (*n* = 8) according to a single study (no data were available for women). Fasting glucose was 99.1 mg/dL (83.7–106.0 mg/dL; *n* = 56) and 3.5 (1.7–4.6; *n* = 20) for the HOMA1. Individuals with motor-incomplete SCI exhibited obesity (BMI) and insulin resistance (HOMA1) but did not meet CMS criteria (Table 2).

Motor-complete Injuries: In individuals with motor-complete SCI, BMI was 24.1 kg/m^2^ (23.7–24.5 kg/m^2^; *n* = 2607), TBFP was 29.3% (27.7–31.2%; *n* = 528) in men and 45.6% (45.5–45.7%; *n* = 20) in women, and visceral fat was 1389.9 mL (841.8–1948.3 mL; *n* = 252). Systolic and diastolic blood pressures were 113.7 mmHg (111.3–116.0 mmHg; *n* = 1025) and 69.6 mmHg (68.2–70.9; *n* = 987), respectively. Triglyceride levels were 120.4 mg/dL (115.8–124.7 mg/dL; *n* = 1607), while in men, HDL-C was 37.3 mg/dL (36.0–38.7; *n* = 747) and 42.7 mg/dL (42.0–43.2; *n* = 21) in women. Fasting glucose levels were 91.4 mg/dL (89.4–93.3 mg/dL; *n* = 1333), 5.4% (5.3–5.5%; *n* = 258) for HbA1c, 0.4 (0.3–0.4; *n* = 47) for QUICKI, and 2.2 (1.8–2.9; *n* = 335) for HOMA1. Men and women with motor-complete SCI meet CMS criteria according to obesity, low HDL-C, and insulin resistance (Table 2).

Low Sympathetic Injury Level: In low sympathetic injury levels, BMI was 25.2 kg/m^2^ (23.6–26.1; *n* = 564) and TBFP was 28.2% (23.4–35.3; *n* = 83) in men and 45.3% (*n* = 6) for women according to one paper. Blood pressures were 126.8 mmHg (122.3–134.0 mmHg; *n* = 261) for systolic and 77.8 mmHg (75.6–79.7 mmHg; *n* = 204) for diastolic. Triglycerides were 137.7 mg/dL (128.1–150.7; *n* = 405), while in men, HDL-C was 40.3 mg/dL (37.2–45.5; *n* = 181) (no data were available for women). Fasting glucose levels were 88.4 mg/dL (85.5–94.2; *n* = 175), HbA1c of 5.5% (4.9–5.6%; *n* = 70), and HOMA1 quantified at 2.3 (1.5–4.6; *n* = 150). Men and women with low sympathetic injuries met the criteria for obesity and insulin resistance (HOMA1) but did not qualify for CMS (Table 2).

High Sympathetic Injury Level: BMI was 24.8 kg/m^2^ (24.3–25.3; *n* = 3194), TBFP of 26.6% (24.2–29.3; *n* = 261) in men (no data were available for women), and visceral fat measuring 1828.9 mL (1166.0–1983.0 mL, *n* = 69). Systolic and diastolic blood pressures were calculated as 111.0 mmHg (107.9–113.8; *n* = 1410) and 68.7 mmHg (66.7–70.3; *n* = 1222), respectively. Triglyceride concentrations were 128.5 mg/dL (121.4–134.5; *n* = 2037), while HDL-C in men was 39.1 mg/dL (36.8–40.8; *n* = 557) and 58.9 mg/dL (58.7–59.0; *n* = 16) in women. Fasting glucose was 96.2 mg/dL (90.8–101.8; *n* = 934), HbA1c measured 5.6% (5.4–5.7; *n* = 405), and HOMA1 was 1.8 (1.5–2.2; *n* = 274). Individuals with high sympathetic injuries exceeded thresholds for obesity and HOMA1. Only men, not women, had low HDL-C. Thus, men with high sympathetic SCI met CMS criteria (Table 2).

Athletes with SCI: Among athletes with SCI, BMI measured 22.4 kg/m^2^ (22.2–22.7; *n* = 610), with a TBFP of 22.5% (20.2–24.7; *n* = 151) in men and 31.9% (*n* = 8) according to a single study in women. Systolic blood pressure was 110.9 mmHg (107.9–114.7; *n* = 280), and diastolic blood pressure was 65.6 mmHg (62.7–69.3; *n* = 280). Triglyceride was measured at 90.4 mg/dL (84.0–101.0; *n* = 277) and HDL-C at 42.1 mg/dL (40.1–46.5; *n* = 134) in men (no data were available for women). Fasting glucose level was 82.9 mg/dL (80.8–86.8; *n* = 157) and a HOMA1 at 2.8 (1.8–3.2; *n* = 111). Athletes with SCI did not meet CMS criteria, exhibiting only insulin resistance (HOMA1) and obesity (Table 2).

### 3.5. Temporal and Geographical Risk Factors of Cardiometabolic Syndrome (Aim 2)

**Figure 3:**
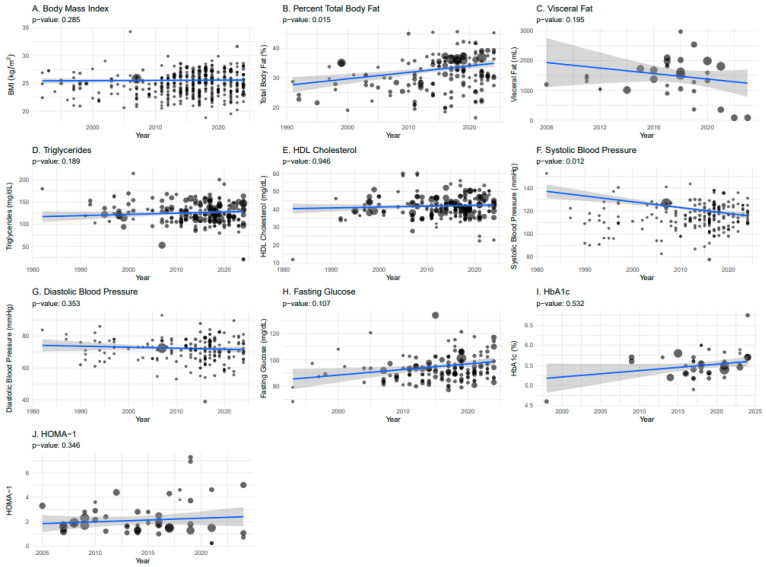
Temporal representation of cardiometabolic syndrome risk factors in the general, non-athletic population of adults with chronic SCI, weighted by sample size and adjusted for publication year, age, weight, proportion of men, and proportion of individuals with tetraplegia. (**A**) body mass index (BMI), (**B**) total body fat percentage, (**C**) visceral fat, (**D**) triglycerides, (**E**) high-density lipoprotein (HDL) cholesterol, (**F**) systolic blood pressure, (**G**) diastolic blood pressure, (**H**) fasting blood glucose, (**I**) Hemoglobin A1C (HbA1c), and (**J**) homeostatic model assessment 1 (HOMA-1) of insulin resistance.

This section presents temporal and geographical trends in CMS component risk factors across the general, non-athletic chronic SCI population, with Figure 3 illustrating their temporal distribution. Most cardiometabolic risk factors remained stable over time, except for a significant increase in TBFP (Figure 3B; *p* = 0.015) and an improvement in systolic blood pressure (Figure 3F; *p* < 0.012).

**Figure 4:**
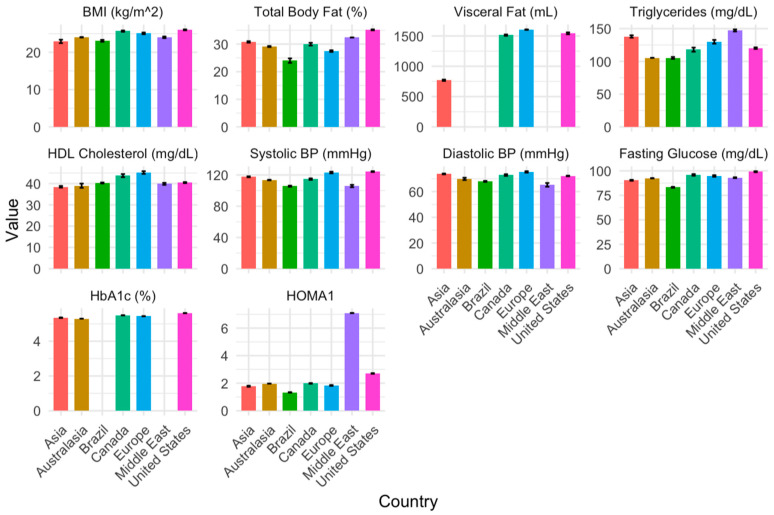
Global representation of cardiometabolic risk factors in the general, non-athletic population of adults with chronic SCI, weighted by study sample size and adjusted for publication year, age, weight, proportion of men, and tetraplegia proportion. Body mass index (BMI), total body fat percentage, visceral fat, triglycerides, high-density lipoprotein (HDL) cholesterol, systolic blood pressure, diastolic blood pressure, fasting blood glucose, Hemoglobin A1C (HbA1c), and homeostatic model assessment 1 (HOMA-1) of insulin resistance.

Overall, 189 studies were conducted in the US (40.1%), 112 in Europe (23.8%), 56 in Canada (11.9%), 54 in Asia (11.5%), 28 in Brazil (5.9%), 17 in Australasia (3.6%), 13 in the Middle East (2.8%), and 2 in South Africa (0.4%). Figure 4 illustrates each CMS risk factor by region. The weighted averages, SE, sample sizes, and references for the risk factors across each geographical area are provided in Supporting Information (Files S10 and S11).

Table 3 presents the β coefficients and corresponding SE values for all cardiometabolic risk factors in each geographical region. Compared to the US, BMI was lower in Asia, Brazil, Europe, and the Middle East (*p* ≤ 0.001). TBFP was lower in Asia, Brazil, Canada, and Europe compared to the US (*p* ≤ 0.003). Visceral fat was lower in Asia than in the US (*p* = 0.007) but similar in Canada and Europe (*p* > 0.05). Compared to the US, triglycerides were greater in Asia, Europe, and the Middle East (*p* < 0.0001). HDL-C was lower in Asia (*p* = 0.037) and greater in Canada and Europe (*p* ≤ 0.001). Systolic and diastolic blood pressures were lower in the Middle East (*p* ≤ 0.007) but higher in Europe (*p* ≤ 0.004) compared to the US. Compared to the US, fasting glucose was lower in Canada and Europe (*p* ≤ 0.028). The Middle East demonstrated higher insulin resistance (*p* < 0.0001) than the US, while Europe demonstrated less (*p* = 0.04). All other findings were similar between regions (*p* > 0.05).

### 3.6. Meta-Analysis of Risk Factors of Cardiometabolic Syndrome (Aim 3)

This section presents Aim 3 findings on the meta-analysis for each subgroup, with results detailed in Table 4, Table 5, Table 6, Table 7 and Table 8.

Persons with vs. without SCI. Table 4 compares cardiometabolic risk and health outcomes between the general, non-athletic population with SCI and controls without SCI. Compared to controls, those with SCI exhibited a greater TBFP and visceral fat with a lower BMI (I^2^ = 62.3–86.0%; *p* < 0.001). Persons with SCI demonstrated lower systolic and diastolic blood pressure than controls (I^2^ = 87.4–90.6%; *p* < 0.001). Triglyceride levels were higher in persons with SCI than controls; however, aggregated HDL-C levels were lower (I^2^ = 79.8–85.3%; *p* ≤ 0.002). Persons with SCI had higher HbA1c (I^2^ = 0.0%) values in comparison to controls (*p* < 0.036). Furthermore, glucose at 120 min during an OGTT (I^2^ = 73.5%) and HOMA1 (I^2^ = 38.5%) were elevated in persons with SCI compared to controls (*p* ≤ 0.024). A complete list of studies included in the meta-analysis comparing the general, non-athletic chronic SCI population with individuals without SCI is available in the Supporting Information (File S12).

Neurological Injury Level. Table 5 compares outcomes between individuals with tetraplegia and paraplegia. BMI was lower in those with tetraplegia (I^2^ = 53.1%; *p* < 0.001), while visceral fat was significantly higher (I^2^ = 0.0%; *p* ≤ 0.015). Compared to paraplegia, tetraplegia was associated with lower systolic and diastolic blood pressure (I^2^ = 83.4–87.8%; *p* < 0.001) and reduced insulin sensitivity and glucose area under the curve (I^2^ = 0.0–36.0%; *p* ≤ 0.03). Meanwhile, individuals with paraplegia had higher HDL-C than those with tetraplegia (I^2^ = 42.0%; *p* < 0.001). A full list of studies included in this neurological level of injury meta-analysis is available in the Supporting Information (File S13).

Injury Completeness. Table 6 presents outcomes for individuals with complete and incomplete SCI, with no significant differences in cardiometabolic risk factors. A full list of studies included in this meta-analysis is available in the Supporting Information (File S14).

Motor-completeness. Table 7 presents cardiometabolic outcomes comparing individuals with motor-complete and motor-incomplete SCI. Those with motor-incomplete SCI had a higher BMI (I^2^ = 49.6; *p* < 0.007) and showed elevated HDL-C (I^2^ = 3.8%) and fasting glucose (I^2^ = 52.7%) compared to individuals with motor-complete injuries (*p* ≤ 0.036). A full list of studies included in this motor completeness meta-analysis is available in the Supporting Information (File S15).

Sympathetic Injury Level. Table 8 presents cardiometabolic risk and health measures in individuals with sympathetic injury levels above and below T6. Those with higher sympathetic injury levels had significantly lower systolic (I^2^ = 62.1%) and diastolic (I^2^ = 24.9%) blood pressures compared to those with lower levels (*p* ≤ 0.001). A full list of studies included in this sympathetic injury level meta-analysis is available in the Supporting Information (File S16).

Athletic Status. Table 9 compares cardiometabolic outcomes between the general, non-athletic SCI population and athletes with SCI. Athletes were heavier than non-athletes (I^2^ = 0.0–46.9%; *p* ≤ 0.03) but had a lower BMI (I^2^ = 23.9%; *p* ≤ 0.001). Systolic (I^2^ = 0.0%) and diastolic (I^2^ = 11.0%) blood pressures were higher in athletes (*p* ≤ 0.002) while fasting glucose levels were lower (I^2^ = 53.2%; *p* < 0.001). A complete list of studies included in this meta-analysis is available in the Supporting Information (File S17).

## 4. Discussion

Our review provides a comprehensive synthesis of evidence on cardiometabolic risk in individuals with chronic SCI. In line with our hypothesis, we observed an injury severity-dependent impact on cardiometabolic health, whereby those with more severe injuries generally exhibited poorer vascular, lipid, and carbohydrate profiles, while athletic status improved health status. We demonstrated that, collectively, men with SCI (overall group, paraplegia, tetraplegia, complete, and high sympathetic injuries) and individuals with motor-complete SCI (men and women) met CMS criteria based on obesity, low HDL-C, and insulin resistance. A further finding was that obesity increased with time, systolic blood pressure improved, and most risk factors remained unchanged. Additionally, specific risk phenotypes are associated with different geographical regions, with the US and other Western regions exhibiting increased severity of cardiometabolic risk factors. Collectively, our findings emphasize the ongoing burden of cardiometabolic risk in chronic SCI, driven by injury severity, regional disparities, and increased obesity, highlighting the evolution of what was once deemed an epidemic [9,10,91] into a global public health pandemic.

### 4.1. Body Composition

After SCI, the loss of dense bone and lean mass, combined with increased adipose tissue, results in a misleadingly lower body weight with an unhealthy fat-to-lean mass ratio. Central obesity and chronic low-grade systemic inflammation are well-documented contributors to cardiovascular risk [12,92]. In our analysis, C-reactive protein (CRP) levels exceeded the >3 mg/L threshold for elevated cardiovascular risk [93], with CRP and tumor necrosis factor-α levels higher in individuals with SCI than in controls. While increased visceral fat likely contributes to this inflammation [94,95,96], adiposity alone does not fully explain the risk profile. Dolbow et al. [97] reported that the fat-to-lean mass ratio was the strongest predictor of metabolic syndrome and systemic inflammation in chronic SCI compared to fat or lean mass alone, emphasizing the crosstalk between these tissues in cardiometabolic risk. Specifically, the loss of skeletal muscle may impair its anti-inflammatory function [98,99], exacerbating systemic inflammation from obesity. Our review highlights that increased total body fat and reduced lean mass may underlie elevated CRP levels and heightened cardiometabolic risk in chronic SCI.

Our findings indicate greater obesity in individuals with tetraplegia compared to those with paraplegia, as evidenced by increased visceral fat, android-to-gynoid ratio, and lower BMI and lean body mass. These results support our hypothesis that injury severity influences myopenia and obesity, leading to a loss of metabolically active lean tissue proportional to injury severity and level. This shift contributes to a new energy requirement “setpoint” post-injury [100]. However, despite these changes in body composition, we found no differences in fat-free mass across comparison groups. In individuals without SCI, body size typically influences energy expenditure through its relationship with fat-free mass, where a larger body size corresponds to greater fat-free mass [101]. Since energy expenditure scales with fat-free mass [102,103] and non-exercising skeletal muscle has minimal impact on this process [104], the primary contributors to variance in energy expenditure are major organs [103,105]. This may explain our findings since organ-related energy dynamics likely remain unchanged after SCI.

### 4.2. Vascular Health

As shown in our study and others, a prominent manifestation of vascular maladaptation after high SCI includes a reduced resting heart rate and blood pressure [72]. Our findings show that individuals with tetraplegia and injuries at or above T6 have lower systolic and diastolic blood pressure than those with paraplegia and injuries below T6. Tetraplegia was also associated with a lower heart rate compared to paraplegia. This finding may reflect the assumption that preganglionic sympathetic cardiac neurons originate from T1–T4/T5. However, evidence suggests variability in cardiac innervation, with contributions from C8 and segments below T5, challenging this view [106] and explaining the lack of heart rate difference between injuries above and below T6. In contrast, peripheral sympathetic control of blood pressure is more widely distributed and less debated [107]. Nonetheless, SCI disrupts descending sympathetic spinal pathways [108,109], leading to autonomic dysfunction, particularly in those with higher-level injuries. This results in cardiovascular instability, including blood pressure fluctuations, bradycardia, hypotension, and autonomic dysreflexia [110]. These fluctuations in blood pressure may predispose individuals with SCI to premature vascular disease and increased cardiovascular risk [111,112,113].

We demonstrated that at both the group and subgroup levels, persons with SCI did not meet the criteria for hypertension. Current risk stratification for hypertension, defined by clinical practice guidelines as ≥130 mmHg systolic or ≥85 mmHg diastolic blood pressure [11], does not consider the injury severity or degree of autonomic dysfunction. Thus, sphygmomanometer-assessed blood pressure may overlook vascular disease, especially when resting pressures fall below conventional thresholds. Our findings contrast with existing reports of hypertension in veterans and civilians with SCI [28,55,114,115,116], many of which do not account for injury level. This discrepancy may be explained by SCI-induced arterial remodeling, which reduces arterial diameter and blood flow, increasing shear stress [117,118]. Tetraplegia also leads to greater carotid intima-media thickness, vascular stiffness, and frequent pressure fluctuations in injuries above T6 [112,119]. However, neglecting injury level and sympathetic dysfunction in blood pressure assessments may overlook vascular pathology, leading to inaccurate risk evaluation in normotensive individuals. This underscores the need for comprehensive vascular assessments, such as ultrasound or ambulatory blood pressure monitoring, and highlights the importance of tailored risk stratification for tetraplegia, as optimal hypertension thresholds may differ from those in paraplegia or individuals without SCI.

### 4.3. Insulin Resistance and Glycemic Health

Reduced physical activity and changes in body composition elevate the risk of insulin resistance in SCI [120]. The notable occurrence of insulin resistance with euglycemic profiles demonstrated in our study signals potential alterations in insulin action in individuals with chronic SCI. These changes likely reflect sympathetic dysregulation [121,122,123] and visceral obesity, especially in those with higher-level injuries. Dysregulation of carbohydrate metabolism is strongly associated with a visceral fat burden [124], which was different based on the neurological level of injury. With abdominal obesity, heightened lipolytic activity in visceral adipocytes results in a flux of non-esterified free fatty acids into the portal circulation, possibly contributing to hepatic and peripheral insulin resistance [9,10]. Due to sublesional myopenia, individuals with SCI have elevated circulating non-esterified free fatty acids compared to those without SCI, as we demonstrated. With limited deposition sites, these fatty acids primarily accumulate in the liver, where they convert to cholesterol and triglycerides [9,10]. Interestingly, individuals with SCI exhibit lower total cholesterol levels than those without SCI but have elevated triglycerides that may be synthesized from the circulating fatty acids. Collectively, these metabolic alterations may sustain insulin resistance in chronic SCI, even in the presence of normal glycemic indices, reflecting a disconnect between glucose appearance and insulin effectiveness. Since direct measurement of insulin resistance is often impractical and unstandardized, fasting plasma glucose is considered a viable clinical alternative; however, glucose alone does not capture insulin action, and the two should be evaluated together to assess carbohydrate metabolism more accurately.

### 4.4. Lipid Health

Several studies have examined lipid profiles in individuals with SCI, consistently reporting low HDL-C concentrations, particularly those with higher injury levels [125,126,127,128,129,130,131,132,133,134]. While dysregulated carbohydrate metabolism is more pronounced in higher injuries, non-HDL-C dyslipidemia is not confined to a specific injury level [40]. Our findings indicate poorer non-HDL-C profiles in individuals with lower and less severe injuries. In contrast, those with higher, more complete injuries exhibit lower HDL-C concentrations, often meeting or falling below the HDL-C threshold associated with increased cardiovascular risk. These disparities likely stem from differences in activity thermogenesis (i.e., physical activity and activities of daily living), which declines with increasing injury severity. Individuals with less severe injuries maintain greater fitness, enabling greater physical activity and energy expenditure, which supports HDL-C production [13,135]. The association between physical activity and HDL-C is attributed to its oxidative stress-reducing and anti-inflammatory properties [136], mediated by enzymes such as lecithin-cholesterol acyltransferase, lipoprotein lipase, and hepatic triglyceride lipase, which regulate HDL-C synthesis, transport, and catabolism [137,138]. Studies in individuals with paraplegia have demonstrated higher HDL-C levels with even modest increases in oxygen consumption during arm ergometry [139]. Thus, insufficient fitness in individuals with SCI may contribute to persistently low HDL-C, restricting its antioxidative and anti-inflammatory functions, particularly in those with higher injuries.

The factors contributing to elevated non-HDL-C profiles in lower injury levels remain unclear despite the better fitness typically associated with these injuries. Two potential explanations are diet [140] and the heterogeneity of lower injuries, spanning thoracic, lumbar, and sacral segments, each with distinct neurological and sympathetic dysfunction [141]. Dietary patterns rich in saturated fats, refined carbohydrates, and added sugars may contribute to the elevated non-HDL-C profiles observed in individuals with lower injuries by stimulating hepatic lipogenesis, increasing VLDL secretion, impairing lipoprotein clearance via reduced lipoprotein lipase activity, and diminishing reverse cholesterol transport, all of which exacerbate dyslipidemia [140]. Although lower injuries are typically associated with better fitness, those involving lower motor neuron damage at or below T9/T10 may present additional barriers to physical activity due to sublesional myopenia, hypotonia, and flaccid paralysis, which may further impair fitness [142,143]. While these factors require further investigation, a healthy lifestyle reduces coronary risk by leveraging its antiatherogenic properties, particularly in higher injury levels. Our findings support this, as athletes with SCI have consistently demonstrated lower cardiometabolic risk profiles than their non-athletic peers. Recent studies also indicate that elite athletes with SCI exhibit superior cardiorespiratory fitness compared to inactive individuals [144], suggesting that greater athletic engagement may confer cardiometabolic benefits in individuals with SCI [13].

### 4.5. Temporal and Geographical Trends

While global cardiovascular mortality trends remain mixed in the population without SCI [145,146,147], risk factors for cardiovascular disease have steadily worsened and are expected to rise further [147,148,149]. Although most risk factors did not worsen in the present review, the lack of improvement is concerning given advancements in medical management, greater awareness, and public health initiatives aimed at reducing cardiovascular disease risk. Notably, our review underscores a substantial increase in the risk factor of obesity, particularly via TBFP. These findings align with global trends in increasing obesity [150]. The rise is likely multifactorial [151], driven by inadequate sleep [152], poor psychosocial health [153,154], urbanization and income status [155,156,157,158,159,160], physical inactivity [161], and the Westernization of energy-dense diets [162,163]. Many of these factors are exacerbated in individuals with SCI, contributing to a worsening health profile [4,64,76,77,78,164,165,166,167,168]. Although systolic blood pressure has decreased (e.g., due to pharmacological or lifestyle management or other unmeasured shifts), suggesting lower hypertensive risk, obesity still confers a silent cardiovascular risk in SCI. Yoon et al. [35] reported that individuals with ‘metabolically healthy obesity’ (SCI-specific BMI > 22 kg/m^2^ with <3 metabolic abnormalities) exhibited increased aortic stiffness despite similar metabolic and inflammatory status, indicating subclinical atherosclerosis even without overt risk factors. This aligns with evidence in non-SCI populations showing that obesity alone increases cardiovascular disease risk relative to someone without obesity [169,170]. These findings highlight the need for proactive cardiovascular monitoring in SCI, even when conventional risk markers appear stable.

Cardiometabolic risk factors in individuals with SCI vary by region, revealing potentially protective and detrimental patterns across different populations. Our regression analysis highlights that obesity (BMI and TBFP) is greater in the US. However, adiposity measures alone do not fully capture metabolic health, as triglyceride levels were higher in Asia, Europe, and the Middle East, suggesting an increased risk of dyslipidemia despite a lower prevalence of obesity in some regions. Addressing cardiometabolic risk in SCI requires a geographically tailored, global strategy, incorporating efforts to curb tobacco and alcohol use, improve diet quality, and promote physical activity. Given its widespread impact, this crisis extends beyond an epidemic, evolving into a global pandemic requiring urgent public health initiatives [171]. Moreover, conventional cardiovascular risk reduction strategies may not fully address SCI-specific metabolic dysfunction, necessitating tailored approaches that consider both neurogenic and regional influences.

#### Strengths and Limitations

Our study provides a comprehensive synthesis of cardiometabolic risk factors in chronic SCI, integrating data from 471 studies to maximize sample sizes. Spanning research from 1981 to 2024, this is the most extensive analysis of metabolic data in SCI, uniquely assessing temporal and geographical trends. Our findings clarify the impact of injury-related factors and athletic activity on cardiometabolic risk. We used dual screening and data extraction in Covidence, examined heterogeneity, and calculated weighted mean differences from a broader dataset than the meta-analysis. This approach strengthens statistical power and precision, yielding robust effect estimates for cardiometabolic risk in the general SCI population and subgroups based on specific injury characteristics.

Several limitations must be considered when interpreting our findings. First, most of the evidence comes from cross-sectional studies, which limit causal inferences due to the inability to establish directionality between SCI and cardiometabolic outcomes. Despite the quality ratings of most studies scoring above 80%, reverse causality remains a potential limitation inherent to cross-sectional designs. Second, pooling data from diverse studies with varying primary outcomes may have introduced heterogeneity. However, we intentionally incorporated multiple risk factor metrics for obesity, dysglycemia, and insulin resistance to enhance sample size and improve risk delineation. Third, we did not differentiate between recreational and elite athletes with SCI or categorize training modalities (resistance vs. aerobic) using objective fitness classifications. Fourth, sympathetic injury classification was based on the T6 level without accounting for autonomic completeness or using a comprehensive autonomic stress test battery. Fifth, due to inconsistent reporting across studies, our review lacked adjustments for physical activity levels, smoking, insurance type, medication use, sleep, and diet. Sixth, our sample was predominantly adult men from Western regions (the US, Canada, and Europe), limiting external validity [172]. However, we provided a large-scale analysis of cardiometabolic risk in women with chronic SCI, a previously underexplored area. Lastly, race and ethnicity, known to influence cardiometabolic risk, were not analyzed [173].

## 5. Conclusions

Expanding on research with a larger cohort, our study minimizes constraints imposed by smaller sample sizes in earlier cardiometabolic health research, allowing us to present nuanced differences from prior studies. Our comprehensive review of individuals with chronic SCI illustrates widespread cardiometabolic risk, meeting two or more risk factors for obesity, low HDL-C, and insulin resistance. Across the SCI subgroups, men with SCI (overall group, paraplegia, tetraplegia, complete, and high sympathetic injuries) and individuals with motor-complete SCI (men and women) met CMS criteria based on obesity, low HDL-C, and insulin resistance. In contrast, although athletes with SCI still show signs of cardiometabolic dysregulation, generally, their risk levels were comparatively lower. While physical activity is cardioprotective, further prevention and management strategies are required. Our findings also illustrate an ongoing increase in the cardiometabolic risk of obesity among individuals with chronic SCI, emphasizing the shift from an epidemic to a global public health pandemic. Addressing the metabolic consequences of SCI is imperative for advancing rehabilitation practices and holds the key to increasing lifespan without disease burden. Distinguishing the relative contributions of neurogenic and lifestyle-related factors to our observed findings remains necessary to establish mechanisms underpinning these metabolic alterations and appropriately tailor countermeasures to injury characteristics in this unique population for future study.

## Figures and Tables

**Table 1:** Cardiometabolic health study outcomes.

Category	Outcomes
Body Composition	Fat: total body, visceral, subcutaneous, upper limb, lower limb, intramuscular, visceral/subcutaneous ratio, and android/gynoid ratio Fat-free mass Lean body mass
Cardiovascular (resting)	Blood pressure: systolic and diastolic Heart rate
Dysglycemia/Insulin Resistance	Fasting glucose, fasting insulin, and hemoglobin A1c OGTT: glucose and insulin area under the curve, peak glucose, glucose at 120 min, and Matsuda index IVGTT: insulin sensitivity and glucose effectiveness HOMA1, HOMA2, and QUICKI
Lipids	Triglycerides
	Cholesterol: total and high-density, low-density, and very low-density lipoprotein
	Non-esterified free fatty acids
Inflammation	C-reactive protein, tumor necrosis factor-α, and interleukin-6

HOMA, Homeostatic model assessment of insulin resistance; IVGTT, intravenous glucose tolerance test; OGTT, oral glucose tolerance test; QUICKI, quantitative insulin sensitivity check index.

**Table 2:** The presence of cardiometabolic risk and cardiometabolic syndrome (CMS) for adults with chronic SCI according to neurological (NLOI) and sympathetic (SLOI) injury levels, injury completeness, and athletic status.

SCI Cohort	Obesity	HTG	Low HDL-C	HTN	Dysglycemia/IR	Risk Factors	CMS
General, non-athletic	✓ (All/♂/♀)	✗	✓ (♂), ✗ (♀)	✗	✓ (All)	3 (♂), 2 (♀)	✓ (♂) ✗ (♀)
Paraplegia NLOI	✓ (All/♂/♀)	✗	✓ (♂), ✗ (♀)	✗	✓ (All)	3 (♂), 2 (♀)	✓ (♂) ✗ (♀)
Tetraplegia NLOI	✓ (All/♂), Ø (♀)	✗	✓ (♂), ✗ (♀)	✗	✓ (All)	3 (♂), 2 (♀)	✓ (♂), Ø (♀)
Incomplete (AIS B-D)	✓ (All), Ø (♀/♂)	✗	✗ (♂), Ø (♀)	✗	✓ (All)	2 (♂), 3 (♀)	✗ (♂), Ø (♀)
Complete (AIS A)	✓ (All/♂), Ø (♀)	✗	✓ (♂), Ø (♀)	✗	✓ (All)	3 (♂), 2 (♀)	✓ (♂), Ø (♀)
Motor-incomplete (AIS C-D)	✓ (All), Ø (♂/♀)	✗	✗ (♂), Ø (♀)	✗	✓ (All)	2 (♂), 2 (♀)	Ø (♂), Ø (♀)
Motor-complete (AIS A-B)	✓ (All/♂/♀)	✗	✓ (♂), ✓ (♀)	✗	✓ (All)	3 (♂), 3 (♀)	✓ (♂), ✓ (♀)
Low SLOI (<T6)	✓ (All/♂/♀)	✗	✗ (♂), Ø (♀)	✗	✓ (All)	2 (♂), 2 (♀)	✗ (♂), Ø (♀)
High SLOI (≥T6)	✓ (All/♂), Ø (♀)	✗	✓ (♂), ✗ (♀)	✗	✓ (All)	3 (♂), 2 (♀)	✓ (♂), ✗ (♀)
Athletes	✓ (All/♂/♀)	✗	✗ (♂), Ø (♀)	✗	✓ (All)	2 (♂), 2 (♀)	✗ (♂), Ø (♀)

Symbol: ✓, presence of a CMS or its risk factor; ✗, no presence of CMS or its risk factors; Ø, unavailable due to insufficient data; ♂, men; ♀, women. AIS, American Spinal Injury Association Impairment Scale; HDL-C, High-density lipoprotein cholesterol; HTG, Hypertriglyceridemia; HTN, Hypertension; IR, Insulin resistance.

**Table 3:** Linear regression analysis of cardiometabolic risk weighted by sample size relative to the United States (the reference population).

	Asia	Australasia	Brazil	Canada	Europe	Middle East
Body mass index	−3.79 (0.21, <0.001)	−1.21 (0.88, 0.171)	−1.88 (0.44, <0.001)	−0.21 (0.25, 0.395)	−1.14 (0.17, <0.001)	−1.55 (0.31, <0.001)
Total body fat percentage	−3.27 (1.08, 0.003)	−2.10 (1.78, 0.240)	−6.45 (1.64, <0.001)	−5.11 (0.82, <0.001)	−5.93 (1.26, <0.001)	2.47 (4.13, 0.551)
Visceral fat	−743.32 (251.53, 0.007)	NA	NA	−455.64 (236.73, 0.066)	78.5 (538.41, 0.885)	NA
Triglycerides	19.85 (5.29, <0.001)	−11.22 (29.80, 0.707)	−10.29 (11.73, 0.382)	6.98 (5.42, 0.199)	12.27 (3.73, 0.001)	29.7 (4.91, <0.001)
HDL-C	−2.23 (1.06, 0.037)	2.03 (3.97, 0.609)	1.46 (1.73, 0.401)	3.42 (1.01, 0.001)	3.49 (0.65, <0.001)	0.01 (1.10, 0.993)
Systolic blood pressure	2.74 (3.27, 0.404)	4.17 (5.34, 0.436)	−4.96 (3.67, 0.178)	−1.02 (2.03, 0.615)	4.36 (1.51, 0.004)	−9.34 (2.68, 0.001)
Diastolic blood pressure	3.68 (2.87, 0.202)	−1.07 (4.45, 0.809)	−1.35 (3.07, 0.661)	2.85 (1.75, 0.105)	5.06 (1.27, 0.000)	−6.16 (2.24, 0.007)
Fasting glucose	−4.02 (2.54, 0.116)	3.68 (6.18, 0.553)	−4.81 (3.36, 0.154)	−4.96 (2.24, 0.028)	−6.00 (1.79, 0.001)	−1.19 (2.37, 0.616)
Hemoglobin A1c	−0.19 (0.15, 0.214)	−0.34 (0.18, 0.067)	NA	−0.08 (0.11, 0.444)	−0.19 (0.10, 0.063)	NA
HOMA1	−0.52 (0.45, 0.256)	−0.19 (0.84, 0.825)	−0.49 (1.18, 0.682)	−0.55 (0.59, 0.358)	−0.89 (0.42, 0.040)	5.24 (1.20, <0.001)

HDL-C, High-density lipoprotein cholesterol; HOMA1, Homeostasis model assessment 1 for insulin resistance. Beta coefficients (standard error, *p*-value) are presented, with significance accepted at *p* ≤ 0.05. Positive β values indicate higher levels of the cardiometabolic risk factor in the specified region compared to the United States. In comparison, negative β values indicate lower levels than those of the United States.

**Table 4:** Meta-analysis summary statistics for cardiometabolic health outcomes for persons with a spinal cord injury (SCI) and healthy controls (HC) without an sci.

	Sample Size (*n*)			WMD	*p*-Value	95% Bootstrap CI		I^2^	Weighted Mean		Weighted SD	
	SCI	HC	Studies			Low	High		SCI	HC	SCI	HC
Body Composition												
Body mass (kg)	2564	2334	106	−3.44	<0.001	−4.68	−2.20	72.99	76.87	79.35	3.87	3.57
Body mass index (kg/m^2^)	3842	3060	87	−0.84	<0.001	−1.26	−0.42	85.96	24.99	26.24	2.17	1.97
Total body fat (kg)	703	884	19	6.13	<0.001	4.49	7.78	79.49	25.94	18.85	3.10	2.61
Total body fat (%)	975	1203	32	7.89	<0.001	6.80	8.99	63.62	32.39	24.95	2.72	2.66
Upper limb fat (%)	43	70	4	6.19	0.031	0.55	11.83	66.63	25.82	22.82	2.98	2.63
Lower limb fat (%)	43	70	4	16.09	<0.001	12.74	19.44	7.66	39.89	27.05	3.13	2.45
Total lean body mass (kg)	405	411	18	−11.40	<0.001	−13.55	−9.24	86.03	43.79	54.68	2.44	2.21
Total fat-free mass (kg)	114	188	6	−4.78	0.195	−12.02	2.45	89.18	54.83	57.53	3.05	2.94
Bone mineral content (kg)	163	133	2	−0.91	0.126	−2.07	0.26	99.64	1.41	2.58	0.62	0.61
Visceral fat (mL)	324	282	5	439.16	<0.001	206.61	671.71	97.13	1645.24	1099.34	28.62	20.14
Subcutaneous fat (mL)	281	246	4	−171.74	0.196	−432.16	88.67	85.02	1280.90	1561.83	27.67	16.98
Visceral fat (%)	134	59	2	3.23	0.220	−1.93	8.39	62.32	11.57	6.59	1.96	1.78
Visceral/subcutaneous fat ratio	115	67	2	0.36	0.106	−0.08	0.80	90.01	1.25	0.66	0.90	0.71
Cardiovascular Health (Resting)												
Systolic blood pressure (mmHg)	1844	1608	57	−7.46	<0.001	−9.48	−5.43	90.64	115.48	123.23	3.86	3.50
Diastolic blood pressure (mmHg)	1637	1506	52	−4.51	<0.001	−5.90	−3.12	87.39	71.08	75.38	3.24	3.03
Heart rate (bpm)	1388	1102	66	1.12	0.099	−0.21	2.45	84.81	70.42	68.51	3.16	3.05
Lipid Metabolism												
Triglycerides (mg/dL)	2370	1420	33	14.53	0.002	5.51	23.54	85.32	118.09	108.50	8.17	7.12
Total cholesterol (mg/dL)	2426	1595	30	−9.33	<0.001	−13.87	−4.78	73.85	182.10	196.30	6.06	6.07
HDL-C (mg/dL)	2564	1592	35	−6.37	<0.001	−7.58	−5.16	79.83	41.05	47.47	3.19	3.31
LDL-C (mg/dL)	2371	1418	31	−1.94	0.289	−5.53	1.65	74.02	116.05	122.89	5.71	5.71
VLDL-C (mg/dL)	112	52	2	4.92	0.374	−5.92	15.75	60.02	19.62	13.37	2.92	3.49
Non-esterified fatty acids (mg/dL)	38	44	5	4.14	<0.001	2.93	5.35	0	36.47	31.80	3.49	4.63
Carbohydrate Metabolism												
Fasting insulin (mU/L)	694	506	22	1.72	0.004	0.53	2.90	84.01	10.20	9.17	2.68	2.36
Fasting glucose (mg/dL)	1122	1027	35	−0.73	0.541	−3.05	1.60	92.65	88.10	92.22	4.37	3.22
Hemoglobin A1C (%)	177	83	4	0.14	0.036	0.01	0.27	0.00	5.42	5.37	1.00	0.76
Glucose AUC during OGTT	44	25	2	106.10	0.230	−67.17	279.37	88.16	489.61	576.34	11.13	10.66
Insulin AUC during OGTT	44	25	2	1863.52	0.331	−1890.78	5617.82	94.00	3602.41	3131.56	42.24	35.66
Glucose at 120 min OGTT	148	98	4	35.59	<0.001	22.73	48.45	73.46	137.68	97.02	6.74	4.69
Matsuda Index OGTT	32	30	2	−2.80	<0.001	−3.64	−1.96	0.00	4.10	6.97	1.25	2.61
HOMA1	337	230	8	0.24	0.0178	0.04	0.44	38.52	1.84	1.58	1.10	0.96
Inflammatory Profile												
Tumor necrosis factor-α (pg/mL)	46	36	4	0.30	0.002	0.11	0.48	0	4.88	5.61	1.74	1.43
Interleukin-6 (mg/mL)	81	67	5	0.61	0.092	−0.10	1.32	23.31	6.29	12.20	2.39	4.18
C-reactive protein (mg/L)	561	557	13	2.03	<0.001	1.03	3.02	92.60	6.68	1.86	3.06	1.43

CI, Confidence Intervals; HDL-C, High-density lipoprotein cholesterol; HOMA1, Homeostatic model assessment 1 insulin resistance; LDL-C, Low-density lipoprotein cholesterol; OGTT, Oral glucose tolerance test; SD, Standard deviation; VLDL-C, Very low-density lipoprotein cholesterol; WMD, Weighted mean difference.

**Table 5:** Meta-analysis summary statistics for cardiometabolic health outcomes by neurological level of injury.

	Sample Size (*n*)			WMD	*p*-Value	95% Bootstrap CI		I^2^	Weighted Mean		Weighted SD	
	Tetra	Para	Studies			Low	High		Tetra	Para	Tetra	Para
Body Composition												
Body mass (kg)	960	1384	49	−1.24	0.070	−2.59	0.10	15.97	75.31	74.92	3.97	3.83
Body mass index (kg/m^2^)	1715	2349	49	−0.90	<0.001	−1.32	−0.47	53.09	24.80	25.36	2.19	2.18
Total body fat (kg)	330	384	14	2.80	0.189	−1.38	6.98	89.44	27.24	24.68	3.05	3.07
Total body fat (%)	435	613	17	0.24	0.749	−1.25	1.74	59.97	33.12	32.17	2.68	2.68
Upper limb fat (%)	95	112	3	2.18	0.456	−3.55	7.91	84.79	28.91	21.21	3.51	3.16
Lower limb fat (%)	81	106	3	0.84	0.608	−2.37	4.05	0	38.38	37.24	2.87	3.55
Total lean body mass (kg)	277	383	14	−3.21	<0.001	−5.04	−1.38	51.38	46.42	48.62	2.92	2.71
Total fat-free mass (kg)	103	119	6	−4.93	0.310	−14.46	4.60	92.86	38.81	45.81	3.13	3.10
Android/gynoid fat (kg)	86	96	3	0.10	0.003	0.03	0.17	0	1.69	1.56	0.76	0.73
Bone mineral content (kg)	149	144	5	−0.06	0.561	−0.27	0.15	65.20	2.08	2.20	0.71	0.67
Visceral fat (mL)	97	139	5	297.82	0.004	93.98	501.66	0	1773.96	1484.42	29.62	28.62
Subcutaneous fat (mL)	26	56	3	−84.84	0.628	−428.37	258.68	18.33	2267.60	2897.98	31.81	38.58
Visceral/subcutaneous fat ratio	26	56	3	0.18	0.059	−0.01	0.36	0	0.79	0.61	0.62	0.63
Cardiovascular Health (Resting)												
Systolic blood pressure (mmHg)	623	701	22	−14.34	<0.001	−17.34	−11.34	87.77	110.42	121.01	3.39	3.40
Diastolic blood pressure (mmHg)	603	682	21	−7.46	<0.001	−9.46	−5.46	83.35	67.46	72.82	2.89	2.99
Heart rate (bpm)	490	445	22	−7.80	<0.001	−11.02	−4.58	93.03	68.93	74.88	2.75	3.00
Lipid Metabolism												
Triglycerides (mg/dL)	1038	1466	24	−5.81	0.211	−14.92	3.29	52.40	125.84	130.70	8.64	8.84
Total cholesterol (mg/dL)	1026	1435	23	−12.51	<0.001	−16.81	−8.20	39.70	175.05	187.29	6.05	6.22
HDL-C (mg/dL)	1038	1466	24	−2.05	<0.001	−3.20	−0.90	42.00	40.04	42.17	3.29	3.25
LDL-C (mg/dL)	996	1417	23	−8.89	<0.001	−12.35	−5.43	29.31	109.98	117.02	5.64	5.82
VLDL-C (mg/dL)	123	215	5	3.44	0.369	−4.07	10.96	84.66	27.45	22.58	3.55	3.28
Carbohydrate Metabolism												
Fasting insulin (mU/L)	152	310	5	0.38	0.263	−0.29	1.06	17.87	9.33	8.28	1.98	2.00
Fasting glucose (mg/dL)	473	783	13	−0.31	0.674	−1.77	1.15	8.99	98.95	95.07	4.76	4.35
Hemoglobin A1C (%)	205	205	5	0.12	0.071	−0.01	0.25	0	5.54	5.54	1.06	0.93
Insulin sensitivity (min^−1^/µU/mL^−1^ × 10^−4^)	20	49	2	−5.91	0.007	−10.23	−1.60	0	3.03	8.66	1.77	4.29
Glucose AUC during OGTT	13	32	2	132.00	0.012	29.48	234.52	35.99	720.08	536.94	10.50	10.87
Insulin AUC during OGTT	13	32	2	36.35	0.380	−44.79	117.49	0	394.08	360.53	9.98	14.38
HOMA1	52	141	2	0.17	0.723	−0.75	1.08	65.80	2.13	1.61	1.35	1.14
Inflammatory Profiles												
Tumor necrosis factor-α (pg/mL)	21	45	2	0.10	0.628	−0.31	0.51	0	5.57	7.01	0.90	0.85
Interleukin-6 (mg/mL)	50	168	4	0.08	0.833	−0.67	0.83	0	4.58	2.95	2.44	1.47
C-reactive protein (mg/L)	119	209	5	−0.23	0.428	−0.81	0.34	4.46	3.27	5.01	1.73	2.57

CI, Confidence Intervals; HDL-C, High-density lipoprotein cholesterol; HOMA1, Homeostatic model assessment 1 insulin resistance; LDL-C, Low-density lipoprotein cholesterol; OGTT, Oral glucose tolerance test; Para, Paraplegia; SD, Standard deviation; Tetra, Tetraplegia; VLDL-C, Very low-density lipoprotein cholesterol; WMD, Weighted mean difference.

**Table 6:** Meta-analysis summary statistics for cardiometabolic health outcomes by complete (C) and incomplete (IC) spinal cord injuries.

	Sample Size			WMD	*p*-Value	95% Bootstrap CI		I^2^	Weighted Mean		Weighted SD	
	C	IC	Studies			Low	High		C	IC	C	IC
Body Composition												
Body mass (kg)	163	88	4	0.37	0.875	−4.27	5.02	21.07	75.07	75.38	4.09	4.01
Body mass index (kg/m^2^)	512	250	3	−0.53	0.412	−1.79	0.74	78.93	24.53	25.50	1.97	2.07
Cardiovascular Health (Resting)												
Systolic BP (mmHg)	23	16	2	−1.48	0.807	−13.32	10.36	0	111.83	113.00	4.43	4.79
Diastolic BP (mmHg)	23	16	2	−0.48	0.916	−9.31	8.35	0	67.91	70.38	3.99	3.88
Heart rate (bpm)	18	18	2	5.05	0.194	−2.57	12.66	32.01	67.67	61.22	3.36	2.95

BP, Blood pressure; CI, Confidence Intervals; Standard deviation; WMD, Weighted mean difference.

**Table 7:** Meta-analysis summary statistics for cardiometabolic health outcomes by motor-complete (MC) and motor-incomplete (MIC) spinal cord injuries.

	Sample Size (*n*)			WMD	*p*-Value	95% Bootstrap CI		I^2^	Weighted Mean		Weighted SD	
	MC	MIC	Studies			Low	High		MC	MIC	MC	MIC
Body Composition												
Body mass (kg)	149	44	2	−2.92	0.362	−9.22	3.37	40.65	75.32	76.01	4.14	3.95
Body mass index (kg/m^2^)	511	132	3	−1.27	0.007	−2.19	−0.34	49.61	24.50	26.13	1.97	2.09
Lipid Metabolism												
Triglycerides (mg/dL)	371	96	2	0.92	0.928	−18.95	20.79	18.72	120.10	126.65	9.40	10.10
Total cholesterol (mg/dL)	371	96	2	−14.93	0.005	−25.29	−4.57	0	188.83	202.21	6.00	6.98
HDL-C (mg/dL)	371	96	2	−6.10	<0.001	−9.36	−2.84	3.79	41.29	47.07	3.47	3.64
LDL-C (mg/dL)	371	96	2	−7.21	0.126	−16.47	2.04	0	123.17	129.33	6.00	6.52
Carbohydrate Metabolism												
Fasting insulin (mU/L)	149	44	2	−0.60	0.0623	−1.24	0.03	0	9.28	9.65	1.10	1.50
Fasting glucose (mg/dL)	149	44	2	−6.91	0.036	−13.38	−0.44	52.70	96.55	103.31	1.41	2.58

CI, Confidence Intervals; HDL-C, High-density lipoprotein cholesterol; LDL-C, Low-density lipoprotein cholesterol; SD, Standard deviation; WMD, Weighted mean difference.

**Table 8:** Meta-analysis summary statistics for cardiometabolic health outcomes by sympathetic level of injury, above (aSLOI) and below (bSLOI) T6.

	Sample Size (*n*)			WMD	*p*-Value	95% Bootstrap CI		I^2^	Weighted Mean		Weighted SD	
	aSLOI	bSLOI	Studies			Low	High		aSLOI	bSLOI	aSLOI	bSLOI
Body Composition												
Body mass (kg)	422	275	17	−1.38	0.219	−3.59	0.82	0	74.71	75.80	4.01	3.93
Body mass index (kg/m^2^)	657	467	14	−0.57	0.153	−1.34	0.21	37.30	24.56	25.30	2.19	2.19
Total body fat (kg)	102	80	3	−3.59	0.205	−9.14	1.97	69.46	24.00	28.36	3.24	3.22
Total body fat (%)	77	47	3	−4.45	0.298	−12.84	3.94	86.84	22.87	27.83	2.83	2.81
Total lean body mass (kg)	90	54	4	−0.69	0.573	−3.08	1.70	0	50.46	49.80	2.70	2.62
Cardiovascular Health (Resting)												
Systolic blood pressure (mmHg)	282	193	10	−16.52	<0.001	−21.68	−11.36	62.13	108.71	125.71	3.99	4.17
Diastolic blood pressure (mmHg)	197	136	8	−7.12	<0.001	−10.59	−3.65	24.93	71.09	78.21	3.46	3.66
Heart rate (bpm)	202	113	10	−5.23	0.085	−11.19	0.73	74.22	73.51	76.50	3.58	3.47
Lipid Metabolism												
Triglycerides (mg/dL)	440	360	6	−7.50	0.625	−37.60	22.59	96.54	127.06	138.21	7.86	8.03
Total cholesterol (mg/dL)	440	360	6	−9.30	0.024	−17.37	−1.24	73.12	176.92	185.75	5.61	5.89
HDL-C (mg/dL)	440	360	6	−0.54	0.260	−1.49	0.40	21.42	38.74	39.65	2.92	2.84
LDL-C (mg/dL)	440	360	6	−5.14	0.086	−11.01	0.73	62.56	110.58	115.45	5.25	5.42
VLDL-C (mg/dL)	52	52	2	−2.56	0.321	−7.63	2.50	0.00	27.70	30.34	3.32	3.86
Carbohydrate Metabolism												
Fasting insulin (mU/L)	165	130	3	0.78	<0.001	0.32	1.24	0.00	10.02	8.84	2.29	1.85
Fasting glucose (mg/dL)	165	130	3	−0.98	0.817	−9.27	7.32	92.69	83.54	84.63	3.49	2.96
HOMA1	165	130	3	0.21	0.336	−0.22	0.64	72.96	1.79	1.52	1.18	0.84
Inflammatory Profile												
C-reactive protein (mg/L)	110	82	2	0.63	0.019	0.10	1.16	0.00	6.36	6.20	2.74	2.47

CI, Confidence Intervals; HDL-C, High-density lipoprotein cholesterol; HOMA1, Homeostatic model assessment 1 insulin resistance; LDL-C, Low-density lipoprotein cholesterol; SD, Standard deviation; VLDL-C, Very low-density lipoprotein cholesterol; WMD, Weighted mean difference.

**Table 9:** Meta-analysis summary statistics for cardiometabolic health outcomes between the general, non-athletic population with chronic spinal cord injury (SCI and athletes with SCI.

	Sample Size (*n*)			WMD	*p*-Value	95% Bootstrap CI		I^2^	Weighted Mean		Weighted SD	
	SCI	Athletes	Studies			Low	High		SCI	Athletes	SCI	Athletes
Body Composition												
Body mass (kg)	134	151	8	4.27	0.037	0.26	8.28	46.90	76.52	71.15	3.58	3.32
Body mass index (kg/m^2^)	177	189	8	1.08	<0.001	0.68	1.48	23.91	23.85	22.52	1.63	1.51
Total body fat (kg)	68	67	3	4.84	<0.001	2.41	7.27	25.42	21.41	16.40	2.22	2.40
Total body fat (%)	63	55	2	5.68	0.224	−3.47	14.84	90.87	23.88	21.24	2.13	2.09
Total fat-free mass (kg)	59	58	2	−0.26	0.931	−6.25	5.73	65.84	60.16	58.50	2.99	2.94
Cardiovascular Health (Resting)												
Systolic blood pressure (mmHg)	121	135	7	−3.45	<0.001	−4.74	−2.16	0	107.53	109.14	3.69	3.44
Diastolic blood pressure (mmHg)	121	135	7	−1.85	0.002	−3.02	−0.68	10.99	67.19	67.29	3.18	2.91
Heart rate (bpm)	133	144	8	7.63	<0.001	6.73	8.54	0	73.93	67.15	3.52	2.78
Lipid Metabolism												
Triglycerides (mg/dL)	50	61	3	6.70	0.419	−9.54	22.93	0	101.61	95.30	7.24	6.19
Total cholesterol (mg/dL)	21	32	2	5.53	0.487	−10.06	21.11	0	168.79	159.22	5.67	5.27
HDL-C (mg/dL)	114	134	6	−0.04	0.942	−1.13	1.05	41.32	39.81	40.99	2.25	2.50
LDL-C (mg/dL)	114	134	6	9.90	<0.001	5.44	14.37	39.03	108.80	97.58	4.50	4.29
Carbohydrate Metabolism												
Fasting glucose (mg/dL)	114	134	6	2.86	<0.001	1.47	4.25	53.19	83.68	82.55	2.53	2.12
Inflammatory Profile												
C-reactive protein (mg/L)	63	78	3	2.38	0.253	−1.70	6.46	97.24	4.26	1.92	4.15	4.00

CI, Confidence Intervals; HDL-C, High-density lipoprotein cholesterol; LDL-C, Low-density lipoprotein cholesterol; SD, Standard deviation; WMD, Weighted mean difference.

## Supplementary Materials

The following supporting information can be downloaded at: https://www.mdpi.com/article/10.3390/jcm14092872/s1, File S1. Title: Systematic review search strategy; File S2. Title: References included in this review; File S3. Title: Quality assessments; File S4. Title: Weighted averages; File S5. Title: Reference for the weighted average; File S6. Title: Weighted averages for men; File S7. Title: References for weighted averages for men; File S8. Title: Weighted averages for women; File S9. Title: References for weighted averages for women; File S10. Title: Weighted averages by geographical region; File S11. Title: References for weighted averages by geographical region; File S12. Title: References in the meta-analysis of SCI and controls; File S13. Title: References in the meta-analysis for the neurological level of injury; File S14. Title: References in the meta-analysis for complete and incomplete SCI; File S15. Title: References included in the meta-analysis for motor-complete and motor-incomplete SCI; File S16. Title: References included in the meta-analysis by the sympathetic level of injury; File S17. Title: References included in the meta-analysis by athlete status.
