# Supplementary material for: Cardiometabolic Risk in Chronic Spinal Cord Injury: A Systematic Review with Meta-Analysis and Temporal and Geographical Trends
Source: J Clin Med. 2025 Apr 22;14(9):2872. doi: 10.3390/jcm14092872 (PMC12072459; doi:10.3390/jcm14092872)
Supplement: Supplementary file 1 [file jcm-14-02872-s001.zip › S1_Systematic review search strategy.v2.pdf]

Supplemental File Containing the Detailed Search Strategy

Ovid MEDLINE(R) and Epub Ahead of Print, In-Process & Other Non-Indexed Citations and Daily 1946 to search date
Searched October 22, 2021. 6983 records retrieved.
Search updated February 15, 2023, 7506 records retrieved, using MEDLINE-ALL segment.
Search updated February 13, 2023, 8019 records retrieved, using MEDLINE-ALL segment.
Search updated September 27, 2024, 8187 records retrieved, using MEDLINE-ALL segment.

1	Spinal Cord Injuries/
2	Paralysis/
3	Paraplegia/
4	exp Quadriplegia/
5	(paralys* or paralyt* or paralyz*).ti,ab,kf.
6	(parapleg* or quadripares* or quadriparet* or quadripleg* or tetrapares* or tetraparet* or tetraplag* or tetrapleg* or quadri-pares* or quadri-paret* or quadri-pleg* or tetra-pares* or tetra-paret* or tetra-plag* or tetra-pleg*).ti,ab,kf.
7	motor-complete.ti,ab,kf.
8	or/1-7
9	exp Spinal Cord/
10	((medulla or medullae) adj3 (spinali or spinalis)).ti,ab,kf.
11	(spinal-cord* or spinalcord* or myelon).ti,ab,kf.
12	((coccygeal or cervical or central or lumbar or sacral or spine or spinal or thoracic) adj3 (cord or cords)).ti,ab,kf.
13	or/9-12
14	Wounds and Injuries/ or Wounds, Nonpenetrating/ or Wounds, Penetrating/ or exp Crush Injuries/
15	Explosions/ or Explosive Agents/ or Blast Injuries/ or Bombs/ or "War-Related Injuries"/ or (battle or battlefield or combat or bomb or bombs or explosion or military or (blast adj2 injur*)).ti,ab,kf.
16	exp Foreign Bodies/ or (foreign adj (body or bodies)).ti,ab,kf.
17	Wounds, Gunshot/ or Firearms/ or (bullet or bullets or firearm or fire-arm or firearms or fire-arms or gunshot or gunshots or gun-shot or gun-shots or gun or guns or shoot or shooting).ti,ab,kf.
18	exp Lacerations/
19	Rupture/
20	Wounds, Stab/
21	(avulsion or avulsions or avulse or avulsed or break or breaks or broke or broken or compress* or pinch* or contuse or contused or contusion or crush or crushed or crushing or damage or damaged or injur* or impale or impaled or impalements or impalement or lacerat* or lesion or lesioned or lesions or perforate or perforated or perforation or post-trauma* or posttrauma* or rupture or ruptured or rupturing or stab or stabbed or stabbing or tear or tears or tore or torn or transect* or trauma* or wound*).ti,ab,kf.
22	injuries.fs.
23	or/14-22
24	13 and 23

25	8 or 24
26	exp Obesity/
27	(obesit* or obese or overweight or over-weight or (adipos* adj3 hyperplasi*) or adiposit* or corpulen* or pickwickian-syndrome).ti,ab,kf.
28	Body Composition/ or Body Fat Distribution/ or Adiposity/
29	((body adj2 mass) or bodymass).ti,ab,kf.
30	exp Body Weight/
31	((body adj2 weight) or bodyweight).ti,ab,kf.
32	Body Mass Index/
33	(body-mass-index or quetelet* index or BMI).ti,ab,kf.
34	Body Height/
35	(height or heights or bodyheight*).ti,ab,kf.
36	exp Adipose Tissue/
37	((fat or adipose or fatty) adj5 (distribution or pattern*)).ti,ab,kf.
38	(four-compartment or 4-compartment).ti,ab,kf.
39	Absorptiometry, Photon/
40	(DXA or DEXA).ti,ab,kf.
41	((((dual-energy or dualenergy or dual-photon) adj1 (x-ray or xray or radiographic)) or (photon or DPX or dual-xray or dual-x-ray or roentgen)) adj1 (absorptiometr* or densitometr* or photodensitometr* or photo-densitometr* or radiodensitometr* or radio-densitometr* or roentgendensimetr* or roentgen-densimetr* or tomodensitometr* or tomo-densitometr*)).ti,ab,kf.
42	((hydrostatic or underwater or hydro-static or under-water) adj1 weigh*).ti,ab,kf.
43	((arm or upper-limb*) adj3 (fat or adipos*)).ti,ab,kf.
44	((leg or lower-limb*) adj3 (fat or adipos*)).ti,ab,kf.
45	Intra-Abdominal Fat/
46	((abdominal or intraabdominal or intra-abdominal or intraperitoneal or intra-peritoneal or organ or retroperitoneal or retro-peritoneal or visceral) adj3 (adipos* or fat or fatty)).ti,ab,kf.
47	Subcutaneous Fat/
48	Subcutaneous Fat, Abdominal/
49	((subcutaneous or abdominal-SC or gluteal or gluteus or buttock*) adj3 (adipos* or fat or fatty)).ti,ab,kf.
50	panniculus adiposus.ti,ab,kf.
51	(VAT adj3 SAT).ti,ab,kf.
52	((intramuscular or intra-muscular or IM) adj3 (adipos* or fat or fatty)).ti,ab,kf.
53	(lean adj3 (mass or weight)).ti,ab,kf.
54	(fat-free adj3 (mass or weight)).ti,ab,kf.
55	Metabolic Syndrome/
56	(cardiometabolic-syndrome* or cardio-metabolic-syndrome* or dysmetabolic-syndrome* or insulin-resistance-syndrome* or metabolic-cardiovascular-syndrome* or metabolic-syndrome* or reaven-syndrome*).ti,ab,kf.
57	Blood Pressure/
58	(((blood or diastolic or pulse or systolic or vascular) adj2 (pressure* or tension)) or normotension).ti,ab,kf.

59	(Pulse/ or Heart Rate/) and rest*.ti,ab,kf.
60	(rest* adj1 (heart-rate* or pulse)).ti,ab,kf.
61	Glucose/
62	Blood Glucose/
63	exp Hyperglycemia/
64	((blood or serum or plasma) adj1 sugar).ti,ab,kf.
65	(glucosaemi$1 or glucosemi$1 or glycaemi$1 or glycemi$1 or hyperglycaemi$1 or hyperglycemia$1 or hyper-glycemia$1 or hyperglycaemi$1 or hyper-glycaemi$1 or 'hyperglucemi$1 or normoglycaemi$1 or normo-glycaemi$1 or normoglycemi$1 or normo-glycemi$1).ti,ab,kf.
66	glucose.ti,ab,kf.
67	Insulin/ or exp "Insulin, Regular, Human"/
68	(insulin or ("actrapid mc" or "humilin" or "iletin" or "immunoinsulin" or "initard" or "insuline" or "insulinum" or "iszilin" or "maxirapid" or "neusulin" or "novolin" or " oralin" or " oro insulin")).ti,ab,kf.
69	(hb-a1c or haemoglobin-a1c or haemoglobin-a-1c or haemoglobin-aic or hba-1c or hba1c or hemoglobin-a1c or hemoglobin-a-1c or hemoglobin-aic).ti,ab,kf.
70	Glycated Hemoglobin A/
71	((glycated or glycosylated) adj3 (hemoglobin* or haemoglobin*)).ti,ab,kf.
72	Insulin Resistance/
73	((insulin or insuline) adj1 (resistanc* or sensitiv* or insensitiv*)).ti,ab,kf.
74	(((homeostasis or homestatic) adj1 model assessment) or HOMA-IR or HOMA-1 or HOMA1 or HOMA-2 or HOMA2).ti,ab,kf.
75	("quantitative insulin sensitivity check index" or quicki).ti,ab,kf.
76	('ogtt' or 'oral-gtt' or "fructosamine test*").ti,ab,kf.
77	Lipids/ or "lipid panel".ti,ab,kf.
78	Cholesterol/ or Cholesterol, HDL/ or Cholesterol, LDL/ or Cholesterol, VLDL/
79	cholesterol*.ti,ab,kf.
80	((high or low) adj1 density-lipoprotein*).ti,ab,kf.
81	Fatty Acids, Nonesterified/
82	((free or nonesterified or non-esterified or unesterified or nonesterized or non-esterized or phosphatide or phospholipid or triglyceride) adj1 fatty-acid*).ti,ab,kf.
83	Cytokines/
84	cytokine*.ti,ab,kf.
85	Adipokines/
86	(adipocytokine* or adipokine*).ti,ab,kf.
87	Adiponectin/
88	(acrp-30 or acrp30 or "adipocyte complement related protein 30" or "adipocyte most abundant protein 1" or adiponectin or adipoq or apm1 or apm-1 or gbp-28 or gbp28 or gelatin binding protein 28).ti,ab,kf.
89	Leptin/
90	(leptin or leptins or ob-gene-product* or ob-protein* or obese-gene-product or obese-protein*).ti,ab,kf.
91	C-Reactive Protein/
92	c-react* protein.ti,ab,kf.

93	Interleukin-6/
94	(b cell stimulat* factor 2 or b lymphocyte stimulating factor 2 or beta 2 interferon or beta2 interferon or hepatocyte stimulating factor or interferon beta 2 or interferon beta2 or interleukin-6 or liver cell stimulating factor or plasmacytoma growth factor or protein 26k or (il-6 or il6 or BSF-2 or IFN-beta 2 or "Interferon beta-2" or MGI-2 or "B-Cell Differentiation Factor" or "Hybridoma Growth Factor")).ti,ab,kf.
95	Interleukin-10/
96	(csif-10 or cytokine synthesis inhibitory factor or il-10 or il10 or interleukin-10).ti,ab,kf.
97	Tumor Necrosis Factor-alpha/
98	(cachectin or cachetin or tumor necrosis factor-alpha or tumour necrosis factor-alpha or tumor necrosis factor-alfa or tumour necrosis factor-alfa or tumor necrosis serum or tumour necrosis serum).ti,ab,kf.
99	or/26-97
100	25 and 99
101	100 not ((exp animals/ not humans/) or (case reports or review).pt.)

Embase (Elsevier, Embase.com)

Sources: Embase, Embase Classic, MEDLINE.

Searched October 22, 2021 5448 records retrieved.

Search updated February 15, 2023, 6357 records retrieved.

Search updated February 13, 2024, 6748 records retrieved.

Search updated September 27, 2024, 6978 records retrieved.

#106	#105 NOT ('case report'/de OR 'review'/it)
#105	#103 NOT #104
#104	('animal'/exp OR 'invertebrate'/exp OR 'animal experiment'/de OR 'animal model'/de OR 'animal tissue'/de OR 'animal cell'/de OR 'nonhuman'/de) NOT (('animal'/exp OR 'invertebrate'/exp OR 'animal experiment'/de OR 'animal model'/de OR 'animal tissue'/de OR 'animal cell'/de OR 'nonhuman'/de) AND ('human'/de OR 'normal human'/de OR 'human cell'/de))
#103	#101 AND #102
#102	#8 AND #21
#101	#22 OR #23 OR #24 OR #25 OR #26 OR #27 OR #28 OR #29 OR #30 OR #31 OR #32 OR #33 OR #34 OR #35 OR #36 OR #37 OR #38 OR #39 OR #40 OR #41 OR #42 OR #43 OR #44 OR #45 OR #46 OR #47 OR #48 OR #49 OR #50 OR #51 OR #52 OR #53 OR #54 OR #55 OR #56 OR #57 OR #58 OR #59 OR #60 OR #61 OR #62 OR #63 OR #64 OR #65 OR #66 OR #67 OR #68 OR #69 OR #70 OR #71 OR #72 OR #73 OR #74 OR #75 OR #76 OR #77 OR #78 OR #79 OR #80 OR #81 OR #82 OR #83 OR #84 OR #85 OR #86 OR #87 OR #88 OR #89 OR #90 OR #91 OR #92 OR #93 OR #94 OR #95 OR #96 OR #97 OR #98 OR #99 OR #100
#100	cachectin:ti,ab,kw OR cachetin:ti,ab,kw OR 'tumor necrosis factor-alpha':ti,ab,kw OR 'tumour necrosis factor-alpha':ti,ab,kw OR 'tumor necrosis factor-alfa':ti,ab,kw OR 'tumour necrosis factor-alfa':ti,ab,kw OR 'tumor necrosis serum':ti,ab,kw OR 'tumour necrosis serum':ti,ab,kw

#99	'tumor necrosis factor'/de
#98	'csif 10':ti,ab,kw OR 'cytokine synthesis inhibitory factor':ti,ab,kw OR 'il 10':ti,ab,kw OR il10:ti,ab,kw OR 'interleukin 10':ti,ab,kw
#97	'interleukin 10'/de
#96	'b cell stimulat* factor 2':ti,ab,kw OR 'b lymphocyte stimulating factor 2':ti,ab,kw OR #2 OR 'beta 2 interferon':ti,ab,kw OR 'beta2 interferon':ti,ab,kw OR 'hepatocyte stimulating factor':ti,ab,kw OR 'interferon beta 2':ti,ab,kw OR 'interferon beta2':ti,ab,kw OR 'interleukin 6':ti,ab,kw OR 'liver cell stimulating factor':ti,ab,kw OR 'plasmacytoma growth factor':ti,ab,kw OR 'protein 26k':ti,ab,kw OR 'il 6':ti,ab,kw OR il6:ti,ab,kw OR 'bsf 2':ti,ab,kw OR 'ifn-beta 2':ti,ab,kw OR 'interferon beta-2':ti,ab,kw OR 'mgi 2':ti,ab,kw OR 'b-cell differentiation factor':ti,ab,kw OR 'hybridoma growth factor':ti,ab,kw
#95	'interleukin 6'/de
#94	'c-react* protein':ti,ab,kw
#93	'c reactive protein'/de
#92	leptin:ti,ab,kw OR leptins:ti,ab,kw OR 'ob gene product*':ti,ab,kw OR 'ob protein*':ti,ab,kw OR 'obese gene product':ti,ab,kw OR 'obese protein*':ti,ab,kw
#91	'leptin'/de
#90	'acrp 30':ti,ab,kw OR acrp30:ti,ab,kw OR 'adipocyte complement related protein 30':ti,ab,kw OR 'adipocyte most abundant protein 1':ti,ab,kw OR adiponectin:ti,ab,kw OR adipoq:ti,ab,kw OR apm1:ti,ab,kw OR 'apm 1':ti,ab,kw OR 'gbp 28':ti,ab,kw OR gbp28:ti,ab,kw OR 'gelatin binding protein 28':ti,ab,kw
#89	'adiponectin'/de
#88	adipocytokine*:ti,ab,kw OR adipokine*:ti,ab,kw
#87	'adipocytokine'/de
#86	cytokine*:ti,ab,kw
#85	'cytokine'/de
#84	((free OR nonesterified OR 'non esterified' OR unesterified OR nonesterized OR 'non esterized' OR phosphatide OR phospholipid OR triglyceride) NEAR/1 'fatty acid*'):ti,ab,kw
#83	'fatty acid'/de
#82	((high OR low) NEAR/1 'density lipoprotein*'):ti,ab,kw
#81	cholesterol*:ti,ab,kw
#80	'cholesterol'/de OR 'high density lipoprotein cholesterol'/de OR 'low density lipoprotein cholesterol'/de OR 'very low density lipoprotein cholesterol'/de
#79	'lipid panel?':ti,ab,kw
#78	'lipid'/de OR 'lipid level'/de
#77	'ogtt':ti,ab,kw OR 'oral-gtt':ti,ab,kw OR 'fructosamine test*':ti,ab,kw
#76	'oral glucose tolerance test'/de
#75	'quantitative insulin sensitivity check index':ti,ab,kw OR quicki:ti,ab,kw
#74	'quantitative insulin sensitivity check index'/de
#73	(((homeostasis OR homestatic) NEAR/1 'model assessment'):ti,ab,kw) OR 'homa ir':ti,ab,kw OR 'homa 1':ti,ab,kw OR homa1:ti,ab,kw OR 'homa 2':ti,ab,kw OR homa2:ti,ab,kw
#72	'homeostasis model assessment'/de

#71	((insulin OR insuline) NEAR/1 (resistanc* OR sensitiv* OR insensitiv*)):ti,ab,kw
#70	'insulin resistance'/de
#69	((glycated OR glycosylated) NEAR/3 (hemoglobin* OR haemoglobin*)):ti,ab,kw
#68	'glycosylated hemoglobin'/exp
#67	'hb a1c':ti,ab,kw OR 'haemoglobin a1c':ti,ab,kw OR 'haemoglobin a 1c':ti,ab,kw OR 'haemoglobin aic':ti,ab,kw OR 'hba 1c':ti,ab,kw OR hba1c:ti,ab,kw OR 'hemoglobin a1c':ti,ab,kw OR 'hemoglobin a 1c':ti,ab,kw OR 'hemoglobin aic':ti,ab,kw
#66	insulin:ti,ab,kw OR 'actrapid mc':ti,ab,kw OR humilin:ti,ab,kw OR iletin:ti,ab,kw OR immunoinsulin:ti,ab,kw OR initard:ti,ab,kw OR insuline:ti,ab,kw OR insulinum:ti,ab,kw OR iszilin:ti,ab,kw OR maxirapid:ti,ab,kw OR neusulin:ti,ab,kw OR novolin:ti,ab,kw OR 'oralin':ti,ab,kw OR 'oro insulin':ti,ab,kw
#65	'insulin'/de OR 'human insulin'/de
#64	glucose:ti,ab,kw
#63	glucosaemi?:ti,ab,kw OR glucosemi?:ti,ab,kw OR glycaemi?:ti,ab,kw OR glycemi?:ti,ab,kw OR hyperglycemia?:ti,ab,kw OR 'hyper glycemia?':ti,ab,kw OR hyperglycaemi?:ti,ab,kw OR 'hyper glycaemi?':ti,ab,kw OR hyperglucemi?:ti,ab,kw OR normoglycaemi?:ti,ab,kw OR 'normo glycaemi?':ti,ab,kw OR normoglycemi?:ti,ab,kw OR 'normo glycemi?':ti,ab,kw
#62	((blood OR serum OR plasma) NEAR/1 sugar):ti,ab,kw
#61	'hyperglycemia'/de
#60	'glucose blood level'/de
#59	'glucose'/de
#58	(rest* NEAR/1 ('heart rate*' OR pulse)):ti,ab,kw
#57	'resting heart rate'/de
#56	(((blood OR diastolic OR pulse OR systolic OR vascular) NEAR/2 (pressure* OR tension)):ti,ab,kw) OR normotension:ti,ab,kw
#55	'blood pressure'/de OR 'diastolic blood pressure'/de OR 'systolic blood pressure'/de
#54	'cardiometabolic syndrome*':ti,ab,kw OR 'cardio metabolic syndrome*':ti,ab,kw OR 'dysmetabolic syndrome*':ti,ab,kw OR 'insulin resistance syndrome*':ti,ab,kw OR 'metabolic cardiovascular syndrome*':ti,ab,kw OR 'metabolic syndrome*':ti,ab,kw OR 'reaven syndrome*':ti,ab,kw
#53	'metabolic syndrome x'/de
#52	(lean NEAR/3 (mass OR weight)):ti,ab,kw
#51	'fat free mass'/de
#50	'lean body weight'/de
#49	((intramuscular OR 'intra muscular' OR im) NEAR/3 (adipos* OR fat OR fatty)):ti,ab,kw
#48	(vat NEAR/3 sat):ti,ab,kw
#47	'panniculus adiposus':ti,ab,kw
#46	((subcutaneous OR 'abdominal sc' OR gluteal OR gluteus OR buttock*) NEAR/3 (adipos* OR fat OR fatty)):ti,ab,kw
#45	'subcutaneous fat'/exp
#44	((abdominal OR intraabdominal OR 'intra abdominal' OR intraperitoneal OR 'intra peritoneal' OR organ OR retroperitoneal OR 'retro peritoneal' OR visceral) NEAR/3 (adipos* OR fat OR fatty)):ti,ab,kw

#43	'intra-abdominal fat'/exp
#42	((skinfold OR 'skin fold') NEXT/3 (thickness* OR measure*)):ti,ab,kw
#41	'skinfold thickness'/de
#40	((leg OR 'lower limb*') NEAR/3 (fat OR adipos*)):ti,ab,kw
#39	((arm OR 'upper limb*') NEAR/3 (fat OR adipos*)):ti,ab,kw
#38	((hydrostatic OR underwater OR 'hydro static' OR 'under water') NEAR/1 weigh*):ti,ab,kw
#37	((photon OR dpx OR 'dual xray' OR 'dual x ray' OR roentgen) NEAR/1 (absorptiometr* OR densitometr* OR photodensitometr* OR 'photo densitometr*' OR radiodensitometr* OR 'radio densitometr*' OR roentgendensimetr* OR 'roentgen densimetr*' OR tomodensitometr* OR 'tomo densitometr*')):ti,ab,kw
#36	(('dual energy' OR dualenergy OR 'dual photon') NEAR/1 ('x ray' OR xray OR radiographic) NEAR/1 (absorptiometr* OR densitometr* OR photodensitometr* OR 'photo densitometr*' OR radiodensitometr* OR 'radio densitometr*' OR roentgendensimetr* OR 'roentgen densimetr*' OR tomodensitometr* OR 'tomo densitometr*')):ti,ab,kw
#35	(('dual energy' OR dualenergy OR 'dual photon') NEAR/1 ('x ray' OR xray OR radiographic)) OR photon OR dpx OR 'dual xray' OR 'dual x ray' OR roentgen
#34	(('dual energy' OR dualenergy OR 'dual photon') NEAR/1 ('x ray' OR xray OR radiographic)) OR photon OR dpx OR 'dual xray' OR 'dual x ray' OR roentgen
#33	'dual energy x ray absorptiometry'/de
#32	'four compartment':ti,ab,kw OR '4 compartment':ti,ab,kw
#31	'compartment model'/de
#30	((fat OR adipose OR fatty) NEAR/5 (distribution OR pattern*)):ti,ab,kw
#29	height:ti,ab,kw OR heights:ti,ab,kw OR bodyheight*:ti,ab,kw
#28	'body mass index':ti,ab,kw OR 'quetelet* index':ti,ab,kw OR bmi:ti,ab,kw
#27	((body NEAR/2 weight):ti,ab,kw) OR bodyweight:ti,ab,kw
#26	((body NEAR/2 mass):ti,ab,kw) OR bodymass:ti,ab,kw
#25	'body mass'/de OR 'body weight'/exp OR 'body height'/de OR 'height'/de
#24	'body composition'/de OR 'body fat distribution'/de OR 'body fat'/de OR 'adipose tissue'/exp OR 'body adiposity index'/de OR 'body distribution'/de OR 'body fat percentage'/de OR 'total body fat'/de
#23	obesit*:ti,ab,kw OR obese:ti,ab,kw OR overweight:ti,ab,kw OR 'over weight':ti,ab,kw OR ((adipos* NEAR/3 hyperplasi*):ti,ab,kw) OR adiposit*:ti,ab,kw OR corpulen*:ti,ab,kw OR 'pickwickian syndrome':ti,ab,kw
#22	'obesity'/exp
#21	#13 AND #20
#20	#14 OR #15 OR #16 OR #17 OR #18 OR #19
#19	avulsion:ti,ab,kw OR avulsions:ti,ab,kw OR avulse:ti,ab,kw OR avulsed:ti,ab,kw OR break:ti,ab,kw OR breaks:ti,ab,kw OR broke:ti,ab,kw OR broken:ti,ab,kw OR compress*:ti,ab,kw OR pinch*:ti,ab,kw OR contuse:ti,ab,kw OR contused:ti,ab,kw OR contusion:ti,ab,kw OR crush:ti,ab,kw OR crushed:ti,ab,kw OR crushing:ti,ab,kw OR damage:ti,ab,kw OR damaged:ti,ab,kw OR injur*:ti,ab,kw OR impale:ti,ab,kw OR impaled:ti,ab,kw OR impalements:ti,ab,kw OR impalement:ti,ab,kw OR lacerat*:ti,ab,kw OR lesion:ti,ab,kw OR lesioned:ti,ab,kw OR lesions:ti,ab,kw OR perforate:ti,ab,kw OR perforated:ti,ab,kw OR perforation:ti,ab,kw OR 'post

	trauma*':ti,ab,kw OR posttrauma*:ti,ab,kw OR rupture:ti,ab,kw OR ruptured:ti,ab,kw OR rupturing:ti,ab,kw OR stab:ti,ab,kw OR stabbed:ti,ab,kw OR stabbing:ti,ab,kw OR tear:ti,ab,kw OR tears:ti,ab,kw OR tore:ti,ab,kw OR torn:ti,ab,kw OR transect*:ti,ab,kw OR trauma*:ti,ab,kw OR wound*:ti,ab,kw
#18	bullet:ti,ab,kw OR bullets:ti,ab,kw OR firearm:ti,ab,kw OR 'fire arm':ti,ab,kw OR firearms:ti,ab,kw OR 'fire arms':ti,ab,kw OR gunshot:ti,ab,kw OR gunshots:ti,ab,kw OR 'gun shot':ti,ab,kw OR 'gun shots':ti,ab,kw OR gun:ti,ab,kw OR guns:ti,ab,kw OR shoot:ti,ab,kw OR shooting:ti,ab,kw
#17	(foreign NEXT/1 (body OR bodies)):ti,ab,kw
#16	battle:ti,ab,kw OR battlefield:ti,ab,kw OR combat:ti,ab,kw OR bomb:ti,ab,kw OR bombs:ti,ab,kw OR explosion:ti,ab,kw OR military:ti,ab,kw OR ((blast NEAR/2 injur*):ti,ab,kw)
#15	'explosive'/exp OR 'bomb'/exp OR 'firearm'/exp OR 'bullet'/de OR 'shrapnel'/de
#14	'injury'/de OR 'battle injury'/de OR 'crush trauma'/exp OR 'foreign body'/de OR 'neck injury'/exp OR 'gunshot injury'/de OR 'knife cut'/de OR 'missile wound'/de OR 'rupture'/exp OR 'shrapnel injury'/de OR 'stab wound'/de
#13	#9 OR #10 OR #11 OR #12
#12	((coccygeal OR cervical OR central OR lumbar OR sacral OR spine OR spinal OR thoracic) NEAR/3 (cord OR cords)):ti,ab,kw
#11	'spinal cord*':ti,ab,kw OR spinalcord*:ti,ab,kw OR myelon:ti,ab,kw
#10	((medulla OR medullae) NEAR/3 (spinali OR spinalis)):ti,ab,kw
#9	'spinal cord'/exp
#8	#1 OR #2 OR #3 OR #4 OR #5 OR #6 OR #7
#7	'motor complete':ti,ab,kw
#6	parapleg*:ti,ab,kw OR quadripares*:ti,ab,kw OR quadriparet*:ti,ab,kw OR quadripleg*:ti,ab,kw OR tetrapares*:ti,ab,kw OR tetraparet*:ti,ab,kw OR tetraplag*:ti,ab,kw OR tetrapleg*:ti,ab,kw OR 'quadri pares*':ti,ab,kw OR 'quadri paret*':ti,ab,kw OR 'quadri pleg*':ti,ab,kw OR 'tetra pares*':ti,ab,kw OR 'tetra paret*':ti,ab,kw OR 'tetra plag*':ti,ab,kw OR 'tetra pleg*':ti,ab,kw
#5	paralys*:ti,ab,kw OR paralyt*:ti,ab,kw OR paralyz*:ti,ab,kw
#4	'quadriplegia'/de
#3	'paraplegia'/de
#2	'paralysis'/de
#1	'spinal cord injury'/de OR 'cervical spinal cord injury'/de OR 'spinal cord compression'/de OR 'spinal cord transsection'/de

Cochrane Central Register of Controlled Trials: CENTRAL (Wiley)
Searched October 22, 2021. 876 records retrieved.
Search updated February 15, 2023. 968 records retrieved.
Search updated February 13, 2024, 1040 records retrieved.
Search updated September 27, 2024, with announcements of trials removed by source-type*, (see line #103), 740 records retrieved.

#1	[mh ^"Spinal Cord Injuries"]
#2	[mh ^Paralysis]
#3	[mh ^Paraplegia]
#4	[mh Quadriplegia]
#5	(paralys*:ti,ab,kw OR paralyt*:ti,ab,kw OR paralyz*:ti,ab,kw)
#6	(parapleg*:ti,ab,kw OR quadripares*:ti,ab,kw OR quadriparet*:ti,ab,kw OR quadripleg*:ti,ab,kw OR tetrapares*:ti,ab,kw OR tetraparet*:ti,ab,kw OR tetraplag*:ti,ab,kw OR tetrapleg*:ti,ab,kw OR quadri-pares*:ti,ab,kw OR quadri-paret*:ti,ab,kw OR quadri-pleg*:ti,ab,kw OR tetra-pares*:ti,ab,kw OR tetra-paret*:ti,ab,kw OR tetra-plag*:ti,ab,kw OR tetra-pleg*:ti,ab,kw)
#7	motor NEXT/1 complete:ti,ab,kw
#8	{OR #1-#7}
#9	[mh "Spinal Cord"]
#10	((medulla:ti,ab,kw OR medullae:ti,ab,kw) NEAR/3 (spinali:ti,ab,kw OR spinalis:ti,ab,kw))
#11	(spinal-cord*:ti,ab,kw OR spinalcord*:ti,ab,kw OR myelon:ti,ab,kw)
#12	((coccygeal:ti,ab,kw OR cervical:ti,ab,kw OR central:ti,ab,kw OR lumbar:ti,ab,kw OR sacral:ti,ab,kw OR spine:ti,ab,kw OR spinal:ti,ab,kw OR thoracic:ti,ab,kw) NEAR/3 (cord:ti,ab,kw OR cords:ti,ab,kw))
#13	{OR #9-#12}
#14	Wounds AND [mh ^Injuries] OR [mh ^"Wounds, Nonpenetrating"] OR [mh ^"Wounds, Penetrating"] OR [mh "Crush Injuries"]
#15	[mh ^Explosions] OR [mh ^"Explosive Agents"] OR [mh ^"Blast Injuries"] OR [mh ^Bombs] OR [mh ^"War-Related Injuries"] OR (battle:ti,ab,kw OR battlefield:ti,ab,kw OR combat:ti,ab,kw OR bomb:ti,ab,kw OR bombs:ti,ab,kw OR explosion:ti,ab,kw OR military:ti,ab,kw OR (blast:ti,ab,kw NEAR/2 injur*:ti,ab,kw))
#16	[mh "Foreign Bodies"] OR (foreign:ti,ab,kw NEXT (body:ti,ab,kw OR bodies:ti,ab,kw))
#17	[mh ^"Wounds, Gunshot"] OR [mh ^Firearms] OR (bullet:ti,ab,kw OR bullets:ti,ab,kw OR firearm:ti,ab,kw OR fire-arm:ti,ab,kw OR firearms:ti,ab,kw OR fire-arms:ti,ab,kw OR gunshot:ti,ab,kw OR gunshots:ti,ab,kw OR gun-shot:ti,ab,kw OR gun-shots:ti,ab,kw OR gun:ti,ab,kw OR guns:ti,ab,kw OR shoot:ti,ab,kw OR shooting:ti,ab,kw)
#18	[mh Lacerations]
#19	[mh ^Rupture]
#20	[mh ^"Wounds, Stab"]

#21	(avulsion:ti,ab,kw OR avulsions:ti,ab,kw OR avulse:ti,ab,kw OR avulsed:ti,ab,kw OR break:ti,ab,kw OR breaks:ti,ab,kw OR broke:ti,ab,kw OR broken:ti,ab,kw OR compress*:ti,ab,kw OR pinch*:ti,ab,kw OR contuse:ti,ab,kw OR contused:ti,ab,kw OR contusion:ti,ab,kw OR crush:ti,ab,kw OR crushed:ti,ab,kw OR crushing:ti,ab,kw OR damage:ti,ab,kw OR damaged:ti,ab,kw OR injur*:ti,ab,kw OR impale:ti,ab,kw OR impaled:ti,ab,kw OR impalements:ti,ab,kw OR impalement:ti,ab,kw OR lacerat*:ti,ab,kw OR lesion:ti,ab,kw OR lesioned:ti,ab,kw OR lesions:ti,ab,kw OR perforate:ti,ab,kw OR perforated:ti,ab,kw OR perforation:ti,ab,kw OR post-trauma*:ti,ab,kw OR posttrauma*:ti,ab,kw OR rupture:ti,ab,kw OR ruptured:ti,ab,kw OR rupturing:ti,ab,kw OR stab:ti,ab,kw OR stabbed:ti,ab,kw OR stabbing:ti,ab,kw OR tear:ti,ab,kw OR tears:ti,ab,kw OR tore:ti,ab,kw OR torn:ti,ab,kw OR transect*:ti,ab,kw OR trauma*:ti,ab,kw OR wound*:ti,ab,kw)
#22	[mh /IN]
#23	{OR #14-#22}
#24	#13 AND #23
#25	#8 OR #24
#26	[mh Obesity]
#27	(obesit*:ti,ab,kw OR obese:ti,ab,kw OR overweight:ti,ab,kw OR over-weight:ti,ab,kw OR (adipos*:ti,ab,kw NEAR/3 hyperplasi*:ti,ab,kw) OR adiposit*:ti,ab,kw OR corpulen*:ti,ab,kw OR pickwickian-syndrome:ti,ab,kw)
#28	[mh ^"Body Composition"] OR [mh ^"Body Fat Distribution"] OR [mh ^Adiposity]
#29	((body:ti,ab,kw NEAR/2 mass:ti,ab,kw) OR bodymass:ti,ab,kw)
#30	[mh "Body Weight"]
#31	((body:ti,ab,kw NEAR/2 weight:ti,ab,kw) OR bodyweight:ti,ab,kw)
#32	[mh ^"Body Mass Index"]
#33	(body-mass-index:ti,ab,kw OR (quetelet* NEXT "index"):ti,ab,kw OR BMI:ti,ab,kw)
#34	(skin NEXT/1 fold or skinfold):ti,ab,kw
#35	[mh ^"Body Height"]
#36	(height:ti,ab,kw OR heights:ti,ab,kw OR bodyheight*:ti,ab,kw)
#37	[mh "Adipose Tissue"]
#38	((fat:ti,ab,kw OR adipose:ti,ab,kw OR fatty:ti,ab,kw) NEAR/5 (distribution:ti,ab,kw OR pattern*:ti,ab,kw))
#39	(four NEXT/1 compartment:ti,ab,kw OR 4 NEXT/1 compartment:ti,ab,kw)
#40	[mh ^"Absorptiometry, Photon"]
#41	(DXA:ti,ab,kw OR DEXA:ti,ab,kw)

#42	((((dual NEXT/1 energy:ti,ab,kw OR dualenergy:ti,ab,kw OR dual NEXT/1 photon:ti,ab,kw) NEAR/1 (x NEXT/1 ray:ti,ab,kw OR xray:ti,ab,kw OR radiographic:ti,ab,kw)) OR (photon:ti,ab,kw OR DPX:ti,ab,kw OR dual NEXT/1 xray:ti,ab,kw OR dual NEXT/1 x NEXT/1 ray:ti,ab,kw OR roentgen:ti,ab,kw)) NEAR/1 (absorptiometr*:ti,ab,kw OR densitometr*:ti,ab,kw OR photodensitometr*:ti,ab,kw OR photo NEXT/1 densitometr*:ti,ab,kw OR radiodensitometr*:ti,ab,kw OR radio NEXT/1 densitometr*:ti,ab,kw OR roentgendensimetr*:ti,ab,kw OR roentgen NEXT/1 densimetr*:ti,ab,kw OR tomodensitometr*:ti,ab,kw OR tomo NEXT/1 densitometr*:ti,ab,kw))
#43	((hydrostatic:ti,ab,kw OR underwater:ti,ab,kw OR hydro-static:ti,ab,kw OR under-water:ti,ab,kw) NEAR/1 weigh*:ti,ab,kw)
#44	((arm:ti,ab,kw OR upper-limb*:ti,ab,kw) NEAR/3 (fat:ti,ab,kw OR adipos*:ti,ab,kw))
#45	((leg:ti,ab,kw OR lower-limb*:ti,ab,kw) NEAR/3 (fat:ti,ab,kw OR adipos*:ti,ab,kw))
#46	[mh ^"Intra-Abdominal Fat"]
#47	((abdominal:ti,ab,kw OR intraabdominal:ti,ab,kw OR intra-abdominal:ti,ab,kw OR intraperitoneal:ti,ab,kw OR intra-peritoneal:ti,ab,kw OR organ:ti,ab,kw OR retroperitoneal:ti,ab,kw OR retro-peritoneal:ti,ab,kw OR visceral:ti,ab,kw) NEAR/3 (adipos*:ti,ab,kw OR fat:ti,ab,kw OR fatty:ti,ab,kw))
#48	[mh ^"Subcutaneous Fat"]
#49	[mh ^"Subcutaneous Fat, Abdominal"]
#50	((subcutaneous:ti,ab,kw OR abdominal-SC:ti,ab,kw OR gluteal:ti,ab,kw OR gluteus:ti,ab,kw OR buttock*:ti,ab,kw) NEAR/3 (adipos*:ti,ab,kw OR fat:ti,ab,kw OR fatty:ti,ab,kw))
#51	panniculus adiposus:ti,ab,kw
#52	(VAT:ti,ab,kw NEAR/3 SAT:ti,ab,kw)
#53	((intramuscular:ti,ab,kw OR intra-muscular:ti,ab,kw OR IM:ti,ab,kw) NEAR/3 (adipos*:ti,ab,kw OR fat:ti,ab,kw OR fatty:ti,ab,kw))
#54	(lean:ti,ab,kw NEAR/3 (mass:ti,ab,kw OR weight:ti,ab,kw))
#55	(fat NEXT/1 free:ti,ab,kw NEAR/3 (mass:ti,ab,kw OR weight:ti,ab,kw))
#56	[mh ^"Metabolic Syndrome"]
#57	(cardiometabolic-syndrome*:ti,ab,kw OR cardio-metabolic-syndrome*:ti,ab,kw OR dysmetabolic-syndrome*:ti,ab,kw OR insulin-resistance-syndrome*:ti,ab,kw OR metabolic-cardiovascular-syndrome*:ti,ab,kw OR metabolic-syndrome*:ti,ab,kw OR reaven-syndrome*:ti,ab,kw)
#58	[mh ^"Blood Pressure"]
#59	(((blood:ti,ab,kw OR diastolic:ti,ab,kw OR pulse:ti,ab,kw OR systolic:ti,ab,kw OR vascular:ti,ab,kw) NEAR/2 (pressure*:ti,ab,kw OR tension:ti,ab,kw)) OR normotension:ti,ab,kw)
#60	([mh ^Pulse] OR [mh ^"Heart Rate"]) AND rest*:ti,ab,kw
#61	(rest*:ti,ab,kw NEXT (heart-rate*:ti,ab,kw OR pulse:ti,ab,kw))
#62	[mh ^Glucose]
#63	[mh ^"Blood Glucose"]

#64	[mh Hyperglycemia]
#65	((blood:ti,ab,kw OR serum:ti,ab,kw OR plasma:ti,ab,kw) NEAR/1 sugar:ti,ab,kw)
#66	(glucosaemi?:ti,ab,kw OR glucosemi?:ti,ab,kw OR glycaemi?:ti,ab,kw OR glycemi?:ti,ab,kw OR hyperglycaemi?:ti,ab,kw OR hyperglycemia?:ti,ab,kw OR hyper-glycemia?:ti,ab,kw OR hyperglycaemi?:ti,ab,kw OR hyper-glycaemi?:ti,ab,kw OR 'hyperglucemi?:ti,ab,kw OR normoglycaemi?:ti,ab,kw OR normo-glycaemi?:ti,ab,kw OR normoglycemi?:ti,ab,kw OR normo-glycemi?:ti,ab,kw)
#67	glucose:ti,ab,kw
#68	[mh ^Insulin] OR [mh "Insulin, Regular, Human"]
#69	(insulin:ti,ab,kw OR ("actrapid mc":ti,ab,kw OR humilin:ti,ab,kw OR iletin:ti,ab,kw OR immunoinsulin:ti,ab,kw OR initard:ti,ab,kw OR insuline:ti,ab,kw OR insulinum:ti,ab,kw OR iszilin:ti,ab,kw OR maxirapid:ti,ab,kw OR neusulin:ti,ab,kw OR novolin:ti,ab,kw OR " oralin":ti,ab,kw OR " oro insulin":ti,ab,kw))
#70	(hb-a1c:ti,ab,kw OR haemoglobin-a1c:ti,ab,kw OR haemoglobin-a-1c:ti,ab,kw OR haemoglobin-aic:ti,ab,kw OR hba-1c:ti,ab,kw OR hba1c:ti,ab,kw OR hemoglobin-a1c:ti,ab,kw OR hemoglobin-a-1c:ti,ab,kw OR hemoglobin-aic:ti,ab,kw)
#71	[mh ^"Glycated Hemoglobin A"]
#72	((glycated:ti,ab,kw OR glycosylated:ti,ab,kw) NEAR/3 (hemoglobin*:ti,ab,kw OR haemoglobin*:ti,ab,kw))
#73	[mh ^"Insulin Resistance"]
#74	((insulin:ti,ab,kw OR insuline:ti,ab,kw) NEXT (resistanc*:ti,ab,kw OR sensitiv*:ti,ab,kw OR insensitiv*:ti,ab,kw))
#75	(((homeostasis:ti,ab,kw OR homestatic:ti,ab,kw) NEXT "model assessment":ti,ab,kw) OR HOMA-IR:ti,ab,kw OR HOMA-1:ti,ab,kw OR HOMA1:ti,ab,kw OR HOMA-2:ti,ab,kw OR HOMA2:ti,ab,kw)
#76	("quantitative insulin sensitivity check index":ti,ab,kw OR quicki:ti,ab,kw)
#77	('ogtt':ti,ab,kw OR 'oral-gtt':ti,ab,kw OR ("fructosamine" NEXT test*):ti,ab,kw)
#78	[mh ^Lipids] OR "lipid panel":ti,ab,kw
#79	[mh ^Cholesterol] OR [mh ^"Cholesterol, HDL"] OR [mh ^"Cholesterol, LDL"] OR [mh ^"Cholesterol, VLDL"]
#80	cholesterol*:ti,ab,kw
#81	((high:ti,ab,kw OR low:ti,ab,kw) NEXT density NEXT lipoprotein*:ti,ab,kw)
#82	[mh ^"Fatty Acids, Nonesterified"]
#83	((free:ti,ab,kw OR nonesterified:ti,ab,kw OR non-esterified:ti,ab,kw OR unesterified:ti,ab,kw OR nonesterized:ti,ab,kw OR non NEXT esterized:ti,ab,kw OR phosphatide:ti,ab,kw OR phospholipid:ti,ab,kw OR triglyceride:ti,ab,kw) NEAR/1 fatty NEXT acid*:ti,ab,kw)
#84	[mh ^Cytokines]
#85	cytokine*:ti,ab,kw

#86	[mh ^Adipokines]
#87	(adipocytokine*:ti,ab,kw OR adipokine*:ti,ab,kw)
#88	[mh ^Adiponectin]
#89	(acrp NEXT 30:ti,ab,kw OR acrp30:ti,ab,kw OR "adipocyte complement related protein 30":ti,ab,kw OR "adipocyte most abundant protein 1":ti,ab,kw OR adiponectin:ti,ab,kw OR adipoq:ti,ab,kw OR apm1:ti,ab,kw OR apm NEXT 1:ti,ab,kw OR gbp-28:ti,ab,kw OR gbp28:ti,ab,kw OR "gelatin binding protein 28":ti,ab,kw)
#90	[mh ^Leptin]
#91	(leptin:ti,ab,kw OR leptins:ti,ab,kw OR ob-gene-product*:ti,ab,kw OR ob-protein*:ti,ab,kw OR obese-gene-product:ti,ab,kw OR obese-protein*:ti,ab,kw)
#92	[mh ^"C-Reactive Protein"]
#93	(c-react* NEXT "protein"):ti,ab,kw
#94	[mh ^Interleukin-6]
#95	((b cell stimulat* factor 2) or (b lymphocyte stimulating factor 2) or (beta 2 interferon) or (beta2 interferon) or (hepatocyte stimulating factor) or (interferon beta 2) or (interferon beta2) or (interleukin NEXT 6) or (liver cell stimulating factor) or (plasmacytoma growth factor) or (protein 26k) or (il NEXT 6) or il6 or (BSF NEXT 2) or (IFN NEXT beta 2) or "Interferon beta-2" or (MGI NEXT 2) or "B-Cell Differentiation Factor" or "Hybridoma Growth Factor"):ti,ab,kw
#96	[mh ^Interleukin-10]
#97	(csif NEXT 10:ti,ab,kw OR "cytokine synthesis inhibitory factor":ti,ab,kw OR il NEXT 10:ti,ab,kw OR il10:ti,ab,kw OR interleukin NEXT 10:ti,ab,kw)
#98	[mh ^"Tumor Necrosis Factor-alpha"]
#99	(cachectin:ti,ab,kw OR cachetin:ti,ab,kw OR "tumor necrosis factor-alpha":ti,ab,kw OR "tumour necrosis factor-alpha":ti,ab,kw OR "tumor necrosis factor-alfa":ti,ab,kw OR "tumour necrosis factor-alfa":ti,ab,kw OR "tumor necrosis serum":ti,ab,kw OR "tumour necrosis serum":ti,ab,kw)
#100	{OR #26-#99}
#101	#25 AND 100
#102	#25 AND 100 in Trials
#103*	#102 NOT (trialsearch:so OR clinicaltrials:so)

CINAHL Plus with Full Text (Ebsco)
Searched October 31, 2021, 3103 records retrieved.
Search updated February 15, 2023, 3319 records retrieved.
Search updated February 13, 2024, 3382 records retrieved.
Search updated September 27, 2024, 3402 records retrieved.

S96	S94 NOT S95
S95	(MH "Animals+") NOT (MH "Human")
S94	S92 NOT S93
S93	(S8 OR S21) AND S91
S92	(S8 OR S21) AND S91
S91	S22 OR S23 OR S24 OR S25 OR S26 OR S27 OR S28 OR S29 OR S30 OR S31 OR S32 OR S33 OR S34 OR S35 OR S36 OR S37 OR S38 OR S39 OR S40 OR S41 OR S42 OR S43 OR S44 OR S45 OR S46 OR S47 OR S48 OR S49 OR S50 OR S51 OR S52 OR S53 OR S54 OR S55 OR S56 OR S57 OR S58 OR S59 OR S60 OR S61 OR S62 OR S63 OR S64 OR S65 OR S66 OR S67 OR S68 OR S69 OR S70 OR S71 OR S72 OR S73 OR S74 OR S75 OR S76 OR S77 OR S78 OR S79 OR S80 OR S81 OR S82 OR S83 OR S84 OR S85 OR S86 OR S87 OR S88 OR S89 OR S90
S90	((TI cachectin OR AB cachectin) OR (TI cachetin OR AB cachetin) OR (TI "tumor necrosis factor-alpha" OR AB "tumor necrosis factor-alpha") OR (TI "tumour necrosis factor-alpha" OR AB "tumour necrosis factor-alpha") OR (TI "tumor necrosis factor-alfa" OR AB "tumor necrosis factor-alfa") OR (TI "tumour necrosis factor-alfa" OR AB "tumour necrosis factor-alfa") OR (TI "tumor necrosis serum" OR AB "tumor necrosis serum") OR (TI "tumour necrosis serum" OR AB "tumour necrosis serum"))
S89	(MH "Tumor Necrosis Factor")
S88	((TI csif-10 OR AB csif-10) OR (TI "cytokine synthesis inhibitory factor" OR AB "cytokine synthesis inhibitory factor") OR (TI il-10 OR AB il-10) OR (TI il10 OR AB il10) OR (TI interleukin-10 OR AB interleukin-10))

S87	(TI "b cell stimulat* factor 2" OR AB "b cell stimulat* factor 2") OR (TI "b lymphocyte stimulating factor" OR AB "b lymphocyte stimulating factor") OR (TI "beta 2 interferon" OR AB "beta 2 interferon") OR (TI "beta2 interferon" OR AB "beta2 interferon") OR (TI "hepatocyte stimulating factor" OR AB "hepatocyte stimulating factor") OR (TI "interferon beta 2" OR AB "interferon beta 2") OR (TI "interferon beta2" OR AB "interferon beta2") OR (TI interleukin-6 OR AB interleukin-6) OR (TI "liver cell stimulating factor" OR AB "liver cell stimulating factor") OR (TI "plasmacytoma growth factor" OR AB "plasmacytoma growth factor") OR (TI "protein 26k" OR AB "protein 26k") OR ((TI "il-6" OR AB "il-6") OR (TI il6 OR AB il6) OR (TI "BSF-2" OR AB "BSF-2") OR (TI "IFN-beta 2" OR AB "IFN-beta 2") OR (TI "Interferon beta-2" OR AB "Interferon beta-2") OR (TI "MGI-2" OR AB "MGI-2") OR (TI "B-Cell Differentiation Factor" OR AB "B-Cell Differentiation Factor") OR (TI "Hybridoma Growth Factor" OR AB "Hybridoma Growth Factor")
S86	(MH "Interleukins")
S85	(TI "c-react* protein" OR AB "c-react* protein")
S84	(MH "C-Reactive Protein")
S83	((TI leptin OR AB leptin) OR (TI leptins OR AB leptins) OR (TI ob-gene-product* OR AB ob-gene-product*) OR (TI ob-protein* OR AB ob-protein*) OR (TI obese-gene-product OR AB obese-gene-product) OR (TI obese-protein* OR AB obese-protein*))
S82	(MH "Leptin")
S81	((TI acrp-30 OR AB acrp-30) OR (TI acrp30 OR AB acrp30) OR (TI "adipocyte complement related protein 30" OR AB "adipocyte complement related protein 30") OR (TI "adipocyte most abundant protein 1" OR AB "adipocyte most abundant protein 1") OR (TI adiponectin OR AB adiponectin) OR (TI adipoq OR AB adipoq) OR (TI apm1 OR AB apm1) OR (TI apm-1 OR AB apm-1) OR (TI gbp-28 OR AB gbp-28) OR (TI gbp28 OR AB gbp28) OR (TI "gelatin binding protein 28" OR AB "gelatin binding protein 28"))
S80	(MH "Adiponectin")
S79	((TI adipocytokine* OR AB adipocytokine*) OR (TI adipokine* OR AB adipokine*))
S78	(MH "Adipokines+")
S77	(TI cytokine* OR AB cytokine*)
S76	(MH "Cytokines")
S75	(((TI free OR AB free) OR (TI nonesterified OR AB nonesterified) OR (TI non-esterified OR AB non-esterified) OR (TI unesterified OR AB unesterified) OR (TI nonesterized OR AB nonesterized) OR (TI non-esterized OR AB non-esterized) OR (TI phosphatide OR AB phosphatide) OR (TI phospholipid OR AB phospholipid) OR (TI triglyceride OR AB triglyceride)) N1 (TI fatty-acid* OR AB fatty-acid*))
S74	(((TI high OR AB high) OR (TI low OR AB low)) N1 (TI density-lipoprotein* OR AB density-lipoprotein*))
S73	(TI cholesterol* OR AB cholesterol*)
S72	(MH "Lipoproteins, HDL Cholesterol") OR (MH "Lipoproteins, LDL Cholesterol")

S71	(MH "Cholesterol+")
S70	(MH Lipids) OR (TI "lipid panel" OR AB "lipid panel")
S69	((TI 'ogtt' OR AB 'ogtt') OR (TI 'oral-gtt' OR AB 'oral-gtt') OR (TI "fructosamine test*" OR AB "fructosamine test*"))
S68	((TI "quantitative insulin sensitivity check index" OR AB "quantitative insulin sensitivity check index") OR (TI quicki OR AB quicki))
S67	((((TI homeostasis OR AB homeostasis) OR (TI homestatic OR AB homestatic)) N1 (TI "model assessment" OR AB "model assessment")) OR (TI HOMA-IR OR AB HOMA-IR) OR (TI HOMA-1 OR AB HOMA-1) OR (TI HOMA1 OR AB HOMA1) OR (TI HOMA-2 OR AB HOMA-2) OR (TI HOMA2 OR AB HOMA2))
S66	(((TI insulin OR AB insulin) OR (TI insuline OR AB insuline)) N1 ((TI resistanc* OR AB resistanc*) OR (TI sensitiv* OR AB sensitiv*) OR (TI insensitiv* OR AB insensitiv*)))
S65	(MH "Insulin Resistance")
S64	(((TI glycated OR AB glycated) OR (TI glycosylated OR AB glycosylated)) N3 ((TI hemoglobin* OR AB hemoglobin*) OR (TI haemoglobin* OR AB haemoglobin*)))
S63	(MH "Hemoglobin A, Glycosylated")
S62	((TI hb-a1c OR AB hb-a1c) OR (TI haemoglobin-a1c OR AB haemoglobin-a1c) OR (TI haemoglobin-a-1c OR AB haemoglobin-a-1c) OR (TI haemoglobin-aic OR AB haemoglobin-aic) OR (TI hba-1c OR AB hba-1c) OR (TI hba1c OR AB hba1c) OR (TI hemoglobin-a1c OR AB hemoglobin-a1c) OR (TI hemoglobin-a-1c OR AB hemoglobin-a-1c) OR (TI hemoglobin-aic OR AB hemoglobin-aic))
S61	((TI insulin OR AB insulin) OR ((TI "actrapid mc" OR AB "actrapid mc") OR (TI humilin OR AB humilin) OR (TI iletin OR AB iletin) OR (TI immunoinsulin OR AB immunoinsulin) OR (TI initard OR AB initard) OR (TI insuline OR AB insuline) OR (TI insulinum OR AB insulinum) OR (TI iszilin OR AB iszilin) OR (TI maxirapid OR AB maxirapid) OR (TI neusulin OR AB neusulin) OR (TI novolin OR AB novolin) OR (TI " oralin" OR AB " oralin") OR (TI " oro insulin" OR AB " oro insulin")))
S60	(MH "Insulin+")
S59	(TI glucose OR AB glucose)
S58	((TI glucosaemi? OR AB glucosaemi?) OR (TI glucosemi? OR AB glucosemi?) OR (TI glycaemi? OR AB glycaemi?) OR (TI glycemi? OR AB glycemi?) OR (TI hyperglycaemi? OR AB hyperglycaemi?) OR (TI hyperglycemia? OR AB hyperglycemia?) OR (TI hyper-glycemia? OR AB hyper-glycemia?) OR (TI hyperglycaemi? OR AB hyperglycaemi?) OR (TI hyper-glycaemi? OR AB hyper-glycaemi?) OR (TI 'hyperglucemi? OR AB 'hyperglucemi?) OR (TI normoglycaemi? OR AB normoglycaemi?) OR (TI normo-glycaemi? OR AB normo-glycaemi?) OR (TI normoglycemi? OR AB normoglycemi?) OR (TI normo-glycemi? OR AB normo-glycemi?))
S57	(((TI blood OR AB blood) OR (TI serum OR AB serum) OR (TI plasma OR AB plasma)) N1 (TI sugar OR AB sugar))
S56	(MH "Hyperglycemia") OR (MH "Glucose Intolerance")

S55	(MH "Glucose") OR (MH "Blood Glucose")
S54	((TI rest* OR AB rest*) N1 ((TI heart-rate* OR AB heart-rate*) OR (TI pulse OR AB pulse)))
S53	((MH "Heart Rate") OR (MH "Pulse+")) AND (TI rest* OR AB rest*))
S52	((((TI blood OR AB blood) OR (TI diastolic OR AB diastolic) OR (TI pulse OR AB pulse) OR (TI systolic OR AB systolic) OR (TI vascular OR AB vascular)) N2 ((TI pressure* OR AB pressure*) OR (TI tension OR AB tension))) OR (TI normotension OR AB normotension))
S51	(MH "Blood Pressure+")
S50	((TI cardiometabolic-syndrome* OR AB cardiometabolic-syndrome*) OR (TI cardio-metabolic-syndrome* OR AB cardio-metabolic-syndrome*) OR (TI dysmetabolic-syndrome* OR AB dysmetabolic-syndrome*) OR (TI insulin-resistance-syndrome* OR AB insulin-resistance-syndrome*) OR (TI metabolic-cardiovascular-syndrome* OR AB metabolic-cardiovascular-syndrome*) OR (TI metabolic-syndrome* OR AB metabolic-syndrome*) OR (TI reaven-syndrome* OR AB reaven-syndrome*))
S49	(MH "Metabolic Syndrome X") OR (MH "Insulin Sensitivity")
S48	((TI fat-free OR AB fat-free) N3 ((TI mass OR AB mass) OR (TI weight OR AB weight)))
S47	(MH "Fat Free Mass")
S46	((TI lean OR AB lean) N3 ((TI mass OR AB mass) OR (TI weight OR AB weight)))
S45	(((TI intramuscular OR AB intramuscular) OR (TI intra-muscular OR AB intra-muscular) OR (TI "IM" OR AB "IM")) N3 ((TI adipos* OR AB adipos*) OR (TI fat OR AB fat) OR (TI fatty OR AB fatty)))
S44	((TI VAT OR AB VAT) N3 (TI SAT OR AB SAT))
S43	(TI "panniculus adiposus" OR AB "panniculus adiposus")
S42	(((TI subcutaneous OR AB subcutaneous) OR (TI abdominal-SC OR AB abdominal-SC) OR (TI gluteal OR AB gluteal) OR (TI gluteus OR AB gluteus) OR (TI buttock* OR AB buttock*)) N3 ((TI adipos* OR AB adipos*) OR (TI fat OR AB fat) OR (TI fatty OR AB fatty)))
S41	(((TI abdominal OR AB abdominal) OR (TI intraabdominal OR AB intraabdominal) OR (TI intra-abdominal OR AB intra-abdominal) OR (TI intraperitoneal OR AB intraperitoneal) OR (TI intra-peritoneal OR AB intra-peritoneal) OR (TI organ OR AB organ) OR (TI retroperitoneal OR AB retroperitoneal) OR (TI retro-peritoneal OR AB retro-peritoneal) OR (TI visceral OR AB visceral)) N3 ((TI adipos* OR AB adipos*) OR (TI fat OR AB fat) OR (TI fatty OR AB fatty)))
S40	(((TI leg OR AB leg) OR (TI lower-limb* OR AB lower-limb*)) N3 ((TI fat OR AB fat) OR (TI adipos* OR AB adipos*)))
S39	(((TI arm OR AB arm) OR (TI upper-limb* OR AB upper-limb*)) N3 ((TI fat OR AB fat) OR (TI adipos* OR AB adipos*)))
S38	(((TI hydrostatic OR AB hydrostatic) OR (TI underwater OR AB underwater) OR (TI hydro-static OR AB hydro-static) OR (TI under-water OR AB under-water)) N1 (TI weigh* OR AB weigh*))

S37	(((((TI dual-energy OR AB dual-energy) OR (TI dualenergy OR AB dualenergy) OR (TI dual-photon OR AB dual-photon)) N1 ((TI x-ray OR AB x-ray) OR (TI xray OR AB xray) OR (TI radiographic OR AB radiographic))) OR ((TI photon OR AB photon) OR (TI DPX OR AB DPX) OR (TI dual-xray OR AB dual-xray) OR (TI dual-x-ray OR AB dual-x-ray) OR (TI roentgen OR AB roentgen))) N1 ((TI absorptiometr* OR AB absorptiometr*) OR (TI densitometr* OR AB densitometr*) OR (TI photodensitometr* OR AB photodensitometr*) OR (TI photo-densitometr* OR AB photo-densitometr*) OR (TI radiodensitometr* OR AB radiodensitometr*) OR (TI radio-densitometr* OR AB radio-densitometr*) OR (TI roentgendensimetr* OR AB roentgendensimetr*) OR (TI roentgen-densimetr* OR AB roentgen-densimetr*) OR (TI tomodensitometr* OR AB tomodensitometr*) OR (TI tomo-densitometr* OR AB tomo-densitometr*)))
S36	((TI DXA OR AB DXA) OR (TI DEXA OR AB DEXA))
S35	(MH "Absorptiometry, Photon")
S34	((TI four-compartment OR AB four-compartment) OR (TI 4-compartment OR AB 4-compartment))
S33	(((TI fat OR AB fat) OR (TI adipose OR AB adipose) OR (TI fatty OR AB fatty)) N5 ((TI distribution OR AB distribution) OR (TI pattern* OR AB pattern*)))
S32	(MH "Adipose Tissue+")
S31	((TI height OR AB height) OR (TI heights OR AB heights) OR (TI bodyheight* OR AB bodyheight*))
S30	(MH "Body Height")
S29	((TI body-mass-index OR AB body-mass-index) OR (TI "quetelet* index" OR AB "quetelet* index") OR (TI BMI OR AB BMI))
S28	(MH "Body Mass Index")
S27	(((TI body OR AB body) N2 (TI weight OR AB weight)) OR (TI bodyweight OR AB bodyweight))
S26	(MH "Body Weight") OR (MH "Body Weight Changes")
S25	(((TI body OR AB body) N2 (TI mass OR AB mass)) OR (TI bodymass OR AB bodymass))
S24	(MH "Body Composition") OR (MH "Adipose Tissue Distribution")
S23	((TI obesit* OR AB obesit*) OR (TI obese OR AB obese) OR (TI overweight OR AB overweight) OR (TI over-weight OR AB over-weight) OR ((TI adipos* OR AB adipos*) N3 (TI hyperplasi* OR AB hyperplasi*)) OR (TI adiposit* OR AB adiposit*) OR (TI corpulen* OR AB corpulen*) OR (TI pickwickian-syndrome OR AB pickwickian-syndrome))
S22	(MH "Obesity+")
S21	S13 AND S20
S20	S14 OR S15 OR S16 OR S17 OR S18 OR S19

S19	(TI avulsion OR AB avulsion) OR (TI avulsions OR AB avulsions) OR (TI avulse OR AB avulse) OR (TI avulsed OR AB avulsed) OR (TI break OR AB break) OR (TI breaks OR AB breaks) OR (TI broke OR AB broke) OR (TI broken OR AB broken) OR (TI compress* OR AB compress*) OR (TI pinch* OR AB pinch*) OR (TI contuse OR AB contuse) OR (TI contused OR AB contused) OR (TI contusion OR AB contusion) OR (TI crush OR AB crush) OR (TI crushed OR AB crushed) OR (TI crushing OR AB crushing) OR (TI damage OR AB damage) OR (TI damaged OR AB damaged) OR (TI injur* OR AB injur*) OR (TI impale OR AB impale) OR (TI impaled OR AB impaled) OR (TI impalements OR AB impalements) OR (TI impalement OR AB impalement) OR (TI lacerat* OR AB lacerat*) OR (TI lesion OR AB lesion) OR (TI lesioned OR AB lesioned) OR (TI lesions OR AB lesions) OR (TI perforate OR AB perforate) OR (TI perforated OR AB perforated) OR (TI perforation OR AB perforation) OR (TI post-trauma* OR AB post-trauma*) OR (TI posttrauma* OR AB posttrauma*) OR (TI rupture OR AB rupture) OR (TI ruptured OR AB ruptured) OR (TI rupturing OR AB rupturing) OR (TI stab OR AB stab) OR (TI stabbed OR AB stabbed) OR (TI stabbing OR AB stabbing) OR (TI tear OR AB tear) OR (TI tears OR AB tears) OR (TI tore OR AB tore) OR (TI torn OR AB torn) OR (TI transect* OR AB transect*) OR (TI trauma* OR AB trauma*) OR (TI wound* OR AB wound*)
S18	((TI bullet OR AB bullet OR SU bullet) OR (TI bullets OR AB bullets OR SU bullets) OR (TI firearm OR AB firearm OR SU firearm) OR (TI fire-arm OR AB fire-arm OR SU fire-arm) OR (TI firearms OR AB firearms OR SU firearms) OR (TI fire-arms OR AB fire-arms OR SU fire-arms) OR (TI gunshot OR AB gunshot OR SU gunshot) OR (TI gunshots OR AB gunshots OR SU gunshots) OR (TI gun-shot OR AB gun-shot OR SU gun-shot) OR (TI gun-shots OR AB gun-shots OR SU gun-shots) OR (TI gun OR AB gun OR SU gun) OR (TI guns OR AB guns OR SU guns) OR (TI shoot OR AB shoot OR SU shoot) OR (TI shooting OR AB shooting OR SU shooting))
S17	((TI foreign OR AB foreign) W1 ((TI body OR AB body) OR (TI bodies OR AB bodies))
S16	(TI battle OR AB battle) OR (TI battlefield OR AB battlefield) OR (TI combat OR AB combat) OR (TI bomb OR AB bomb) OR (TI bombs OR AB bombs) OR (TI explosion OR AB explosion) OR (TI military OR AB military) OR ((TI blast OR AB blast) N2 (TI injur* OR AB injur*))
S15	(MH "Firearms+") OR (MH "Gun Violence")
S14	(MH "Wounds and Injuries") OR (MH "Crush Injuries") OR (MH "Foreign Bodies") OR (MH "Athletic Injuries+") OR (MH "Back Injuries+") OR (MH "Blast Injuries") OR (MH "Neck Injuries+") OR (MH "Shock, Traumatic") OR (MH "Rupture") OR (MH "Tears and Lacerations") OR (MH "Wounds, Nonpenetrating") OR (MH "Wounds, Penetrating+")
S13	S9 OR S10 OR S11 OR S12

S12	TI ((coccygeal OR cervical OR lumbar OR sacral OR spine OR spinal OR thoracic) N3 (cord or cords)) OR AB ((coccygeal OR cervical OR lumbar OR sacral OR spine OR spinal OR thoracic) N3 (cord or cords))
S11	TI (spinal-cord* OR spinalcord* OR myelon) OR AB (spinal-cord* OR spinalcord* OR myelon)
S10	TI ((medulla OR medullae) N3 (spinali OR spinalis)) OR AB ((medulla OR medullae) N3 (spinali OR spinalis))
S9	(MH "Spinal Cord+")
S8	S1 OR S2 OR S3 OR S4 OR S5 OR S6 OR S7
S7	TI motor-complete OR AB motor-complete
S6	TI (parapleg* or quadripares* or quadriparet* or quadripleg* or tetrapares* or tetraparet* or tetraplag* or tetrapleg* or quadri-pares* or quadri-paret* or quadri-pleg* or tetra-pares* or tetra-paret* or tetra-plag* or tetra-pleg*) OR AB(parapleg* or quadripares* or quadriparet* or quadripleg* or tetrapares* or tetraparet* or tetraplag* or tetrapleg* or quadri-pares* or quadri-paret* or quadri-pleg* or tetra-pares* or tetra-paret* or tetra-plag* or tetra-pleg*)
S5	TI (paralys* or paralyt* or paralyz*) OR AB (paralys* or paralyt* or paralyz*)
S4	(MH "Quadriplegia+")
S3	(MH "Paraplegia")
S2	(MH "Paralysis")
S1	(MH "Spinal Cord Injuries")

Scopus (Elsevier)
Searched November 11, 2021, 7897 records retrieved.
Search updated February 15, 2023, 8585 records retrieved.
Search updated February 13, 2024, 9454 records retrieved.
Search updated September 27, 2024, 9494 records retrieved.

((((TITLE-ABS(paralys* OR paralyt* OR paralyz* OR parapleg* OR quadripares* OR quadriparet* OR quadripleg* OR tetrapares* OR tetraparet* OR tetraplag* OR tetrapleg* OR quadri-pares* OR quadri-paret* OR quadri-pleg* OR tetra-pares* OR tetra-paret* OR tetra-plag* OR tetra-pleg* OR motor-complete)) OR (TITLE-ABS(((medulla OR medullae) W/3 (spinali OR spinalis)) OR (spinal-cord* OR spinalcord* OR myelon) OR ((coccygeal OR cervical OR central OR lumbar OR sacral OR spine OR spinal OR thoracic) W/3 (cord OR cords)))) AND (TITLE-ABS(battle OR battlefield OR combat OR bomb OR bombs OR explosion OR military OR (blast W/2 injur*) OR (foreign W/1 (body OR bodies)) OR bullet OR bullets OR firearm OR fire-arm OR firearms OR fire-arms OR gunshot OR gunshots OR gun-shot OR gun-shots OR gun OR guns OR shoot OR shooting OR avulsion OR avulsions OR avulse OR avulsed OR break OR breaks OR broke OR broken OR compress* OR pinch* OR contuse OR contused OR contusion OR crush OR crushed OR crushing OR damage OR damaged OR injur* OR impale OR impaled OR impalements OR impalement OR lacerat* OR lesion OR lesioned OR lesions OR perforate OR perforated OR perforation OR post-trauma* OR

posttrauma* OR rupture OR ruptured OR rupturing OR stab OR stabbed OR stabbing OR tear OR tears OR tore OR torn OR transect* OR trauma* OR wound*))) AND (TITLE-ABS (obesit* OR obese OR overweight OR over-weight OR (adipos* W/3 hyperplasi*) OR adiposit* OR corpulen* OR "pickwickian syndrome" OR (body W/2 mass) OR bodymass OR (body W/2 weight) OR bodyweight OR "body mass index" OR "quetelet index" OR BMI OR ((skinfold OR "skin fold") W/1 test) OR height OR heights OR bodyheight* OR ((fat OR adipose OR fatty) W/5 (distribution OR pattern*)) OR "four compartment" OR "4 compartment" OR DXA OR DEXA OR ((((dual-energy OR dualenergy OR dual-photon) W/1 (x-ray OR xray OR radiographic)) OR (photon OR DPX OR dual-xray OR dual-x-ray OR roentgen)) W/1 (absorptiometr* OR densitometr* OR photodensitometr* OR photo-densitometr* OR radiodensitometr* OR radio-densitometr* OR roentgendensimetr* OR roentgen-densimetr* OR tomodensitometr* OR tomo-densitometr*)) OR ((hydrostatic OR underwater OR hydro-static OR under-water) W/1 weigh*) OR ((arm OR upper-limb*) W/3 (fat OR adipos*)) OR ((leg OR lower-limb*) W/3 (fat OR adipos*)) OR ((abdominal OR intraabdominal OR "intra abdominal" OR intraperitoneal OR "intra peritoneal" OR organ OR retroperitoneal OR "retro peritoneal" OR visceral) W/3 (adipos* OR fat OR fatty)) OR ((subcutaneous OR "abdominal SC" OR gluteal OR gluteus OR buttock*) W/3 (adipos* OR fat OR fatty)) OR "panniculus adiposus" OR (VAT W/3 SAT) OR ((intramuscular OR intra-muscular OR IM) W/3 (adipos* OR fat OR fatty)) OR (lean W/3 (mass OR weight)) OR (fat-free W/3 (mass OR weight)) OR "cardiometabolic syndrome" OR "cardio-metabolic syndrome" OR "dysmetabolic syndrome" OR "insulin resistance syndrome" OR "metabolic cardiovascular syndrome" OR "metabolic syndrome" OR "reaven syndrome" OR ((blood OR diastolic OR pulse OR systolic OR vascular) W/2 (pressure* OR tension)) OR normotension OR (rest* W/1 (heart-rate* OR pulse)) OR ((blood OR serum OR plasma) W/1 sugar) OR glucosaemia OR glucosaemic OR glucosemia OR glucosemic OR glycaemia OR glycaemic OR glycemia OR glycemic OR hyperglucemia OR hyperglucemic OR hyperglycaemia OR hyperglycaemia OR "hyper glycaemia" OR hyperglycaemic OR hyperglycaemic OR "hyper glycaemic" OR hyperglycemia OR "hyper glycemia" OR hyperglycemic OR "hyper glycemic" OR normoglycaemia OR "normo glycaemia" OR normoglycaemic OR "normo glycaemic" OR "normo glycemia" OR normoglycemia OR "normo glycemic" OR normoglycemic OR glucose OR insulin OR "actrapid mc" OR humilin OR iletin OR immunoinsulin OR initard OR insuline OR insulinum OR iszilin OR maxirapid OR neusulin OR novolin OR oralin OR "oro insulin" OR {hb-a1c} OR {hb a1c} OR {haemoglobin-a1c} OR {haemoglobin-a1c} OR {haemoglobin-a-1c} OR {haemoglobin-aic} OR {hba-1c} OR hba1c OR {hemoglobin a1c} OR {hemoglobin a 1c} OR {hemoglobin aic} OR ((glycated OR glycosylated) W/3 (hemoglobin* OR haemoglobin*)) OR ((insulin OR insuline) W/1 (resistanc* OR sensitiv* OR insensitiv*)) OR ((homeostasis OR homestatic) W/1 "model assessment") OR HOMA-IR OR {HOMA-1} OR {HOMA 1} OR HOMA1 OR {HOMA-2} OR {HOMA2} OR HOMA2 OR "quantitative insulin sensitivity check index" OR quicki OR ogtt OR "oral gtt" OR "fructosamine test" OR "lipid panel" OR cholesterol* OR ((high OR low) W/1 "density lipoprotein") OR ((free OR nonesterified OR non-esterified OR unesterified OR nonesterized OR "non esterized" OR phosphatide OR phospholipid OR triglyceride) W/1 "fatty acid") OR cytokine* OR adipocytokine* OR adipokine* OR {acrp 30} OR {acrp-30} OR acrp30 OR "adipocyte complement related protein 30" OR "adipocyte most

abundant protein 1" OR adiponectin OR adipoq OR apm1 OR {apm 1} OR {apm-1} OR {gbp 28} OR {gbp-28} OR gbp28 OR "gelatin binding protein 28" OR leptin OR leptins OR "ob-gene-product" OR "ob-protein" OR "obese-gene-product" OR "obese-protein" OR (c-react* w/1 protein*) OR "b cell stimulating factor 2" OR "b lymphocyte stimulating factor 2" OR "beta 2 interferon" OR "beta2 interferon" OR "hepatocyte stimulating factor" OR "interferon beta 2" OR "interferon beta2" OR "interleukin 6" OR "liver cell stimulating factor" OR "plasmacytoma growth factor" OR {protein 26k} OR {protein-26k} OR {il 6} OR {il-6} OR il6 OR {BSF 2} OR {BSF-2} OR {IFN beta 2} OR {IFN-beta-2} OR {IFN beta-2} OR {IFN-beta 2} OR "Interferon beta 2" OR {MGI 2} OR {MGI-2} OR "B Cell Differentiation Factor" OR "Hybridoma Growth Factor" OR {csif 10} OR {csif-10} OR "cytokine synthesis inhibitory factor" OR {il 10} OR {il-10} OR il10 OR "interleukin 10" OR cachectin OR cachetin OR "tumor necrosis factor alpha" OR "tumour necrosis factor alpha" OR "tumor necrosis factor alfa" OR "tumour necrosis factor alfa" OR "tumor necrosis serum" OR "tumour necrosis serum"))) AND NOT (TITLE(animal* OR nonhuman* OR veterinar* OR avian* OR baboon* OR bird* OR bovine OR canine OR cat OR cats OR cattle* OR chick* OR chimp* OR cow OR cows OR dog OR dogs OR duck OR feline OR fish* OR geese OR goose OR macaque* OR marmoset* OR mice OR mouse OR murine OR ovine OR pig OR pigs OR piglet* OR porcine OR primate* OR rabbit OR rat OR rats OR rodent* OR sheep OR swine OR trout* OR zebrafish*) AND NOT (human* OR patient* OR women OR woman OR men OR man))) AND NOT (TITLE ({case report})) AND (LIMIT-TO (DOCTYPE,"ar") OR LIMIT-TO (DOCTYPE,"cp"))

Web of Science (Clarivate)
Searched November 15, 2021. 1437 records retrieved.
Search updated February 15, 2023, 1957 records retrieved.
Search updated February 13, 2024, 2012 records retrieved.
Search updated September 27, 2024, 2056 records retrieved.

Databases searched: *Web of Science Core Collection*: Science Citation Index Expanded (1945-present), Social Sciences Citation Index (1956-present), Arts & Humanities Citation Index (1975-present), Conference Proceedings Citation Index- Science (1990-present), Conference Proceedings Citation Index- Social Science & Humanities (1990-present), Emerging Sources Citation Index (2015-present).
Regional databases: KCI-Korean Journal Database, 1980-present; SciELO Citation Index, 2002-present; Russian Science Citation Index, 2015-present; (2021 search only)), Scientific Electronic Library Online (SciELO) Citation Index (inception to present).
Timespan=1945-date of search
Language = Auto

#56	#55 AND #13 AND #4
#55	#14 or #15 or #16 or #17 or #18 or #19 or #20 or #21 or #22 or #23 or #24 or #25 or #26 or #27 or #28 or #29 or #30 or #31 or #32 or #33 or #34 or #35 or #36 or #37 or #38 or #39 or #40 or #41 or #42 or #43 or #44 or #45 or #46 or #47 or #48 or #49 or #50 or #51 or #52 or #53 or #54
#54	TS=(cachectin or cachetin or tumor necrosis factor$alpha or tumour necrosis factor$alpha or tumor necrosis factor$alfa or tumour necrosis factor$alfa or tumor necrosis serum or tumour necrosis serum)
#53	TS=(csif$10 or cytokine synthesis inhibitory factor or il$10 or il10 or interleukin$10)
#52	TS=(b cell stimulat* factor 2 or b lymphocyte stimulating factor 2 or beta 2 interferon or beta2 interferon or hepatocyte stimulating factor or interferon beta 2 or interferon beta2 or interleukin$6 or liver cell stimulating factor or plasmacytoma growth factor or protein 26k or (il$6 or il6 or BSF$2 or IFN$beta 2 or "Interferon beta$2" or MGI$2 or "B$Cell Differentiation Factor" or "Hybridoma Growth Factor"))
#51	TS=(c$react* protein)
#50	TS=(leptin or leptins or ob$gene-product* or ob$protein* or obese$gene-product or obese$protein*)
#49	TS=(acrp$30 or acrp30 or "adipocyte complement related protein 30" or "adipocyte most abundant protein 1" or adiponectin or adipoq or apm1 or apm$1 or gbp$28 or gbp28 or gelatin binding protein$28)
#48	TS=(adipocytokine* or adipokine*)
#47	TS=((free or nonesterified or non$esterified or unesterified or nonesterized or non$esterized or phosphatide or phospholipid or triglyceride) NEAR/1 fatty$acid*)
#46	TS=((high or low) NEAR/1 density$lipoprotein*)
#45	TS=("lipid panel*")
#44	TS=(cholesterol*)
#43	TS=(ogtt or oral$gtt or "fructosamine test*")
#42	TS=("quantitative insulin sensitivity check index" or quicki)
#41	TS=(((homeostasis or homestatic) NEAR/1 model assessment) or HOMA$IR or HOMA$1 or HOMA1 or HOMA$2 or HOMA2)
#40	TS=((insulin or insuline) NEAR/1 (resistanc* or sensitiv* or insensitiv*))
#39	TS=((glycated or glycosylated) NEAR/3 (hemoglobin* or haemoglobin*))
#38	TS=((hb$a1c or haemoglobin$a1c or haemoglobin a$1c or haemoglobin$aic or hba$1c or hba1c or hemoglobin$a1c or hemoglobin a$1c or hemoglobin$aic))

#37	TS=(insulin or "actrapid mc" or "humilin" or "iletin" or "immunoinsulin" or "initard" or "insuline" or "insulinum" or "iszilin" or "maxirapid" or "neusulin" or "novolin" or "oralin" or "oro insulin")
#36	TS=(glucose)
#35	TS=(glucosaemi* or glucosemi* or glycaemi* or glycemi* or hyperglycaemi* or hyperglycemia* or hyper$glycemia* or hyperglycaemi* or hyper$glycaemi* or 'hyperglucemi* or normoglycaemi* or normo$glycaemi* or normoglycemi* or normo$glycemi*)
#34	TS=((blood or serum or plasma) NEAR/1 sugar)
#33	TS=(((blood or diastolic or pulse or systolic or vascular) NEAR/2 (pressure* or tension)) or normotension)
#32	TS=(cardiometabolic$syndrome* or cardio$metabolic-syndrome* or dysmetabolic$syndrome* or insulin$resistance-syndrome* or metabolic$cardiovascular-syndrome* or metabolic$syndrome* or reaven$syndrome*)
#31	TS=(fat-free NEAR/3 (mass or weight))
#30	TS=(lean NEAR/3 (mass or weight))
#29	TS=((intramuscular or intra$muscular or IM) NEAR/3 (adipos* or fat or fatty))
#28	TS=(VAT NEAR/3 SAT)
#27	TS=(panniculus adiposus)
#26	TS=((subcutaneous or abdominal$SC or gluteal or gluteus or buttock*) NEAR/3 (adipos* or fat or fatty))
#25	TS=((abdominal or intraabdominal or intra$abdominal or intraperitoneal or intra-peritoneal or organ or retroperitoneal or retro$peritoneal or visceral) NEAR/3 (adipos* or fat or fatty))
#24	TS=((leg or lower-limb*) NEAR/3 (fat or adipos*))
#23	TS=((arm or upper-limb*) NEAR/3 (fat or adipos*))
#22	TS=((hydrostatic or underwater or hydro-static or under-water) NEAR/1 weigh*)
#21	TS=((((dual$energy or dualenergy or dual$photon) NEAR/1 (x$ray or xray or radiographic)) or (photon or DPX or dual$xray or dual-x$ray or roentgen)) NEAR/1 (absorptiometr* or densitometr* or photodensitometr* or photo$densitometr* or radiodensitometr* or radio$densitometr* or roentgendensimetr* or roentgen$densimetr* or tomodensitometr* or tomo$densitometr*))
#20	TS=(DXA or DEXA)
#19	TS=(four-compartment or 4-compartment)
#18	TS=((fat or adipose or fatty) NEAR/5 (distribution or pattern*))
#17	TS=(height or heights or bodyheight*)
#16	TS=(body-mass-index or quetelet* index or BMI)
#15	TS=((body NEAR/2 mass) or bodymass)

#14	TS=(obesit* or obese or overweight or over-weight or (adipos* NEAR/3 hyperplasi*) or adiposit* or corpulen* or pickwickian-syndrome)
#13	#12 AND #8
#12	#9 OR #10 OR #11
#11	TS=(bullet or bullets or firearm or fire-arm or firearms or fire-arms or gunshot or gunshots or gun-shot or gun-shots or gun or guns or shoot or shooting)
#10	TS=(battle or battlefield or combat or bomb or bombs or explosion or military or (blast NEAR/2 injur*))
#9	TS=(avulsion or avulsions or avulse or avulsed or break or breaks or broke or broken or compress* or pinch* or contuse or contused or contusion or crush or crushed or crushing or damage or damaged or injur* or impale or impaled or impalements or impalement or lacerat* or lesion or lesioned or lesions or perforate or perforated or perforation or post-trauma* or posttrauma* or rupture or ruptured or rupturing or stab or stabbed or stabbing or tear or tears or tore or torn or transect* or trauma* or wound*)
#8	#5 OR #6 OR #7
#7	TS=((coccygeal or cervical or central or lumbar or sacral or spine or spinal or thoracic) NEAR/3 (cord or cords))
#6	TS=(spinal-cord* or spinalcord* or myelon)
#5	TS=((medulla or medullae) NEAR/3 (spinali or spinalis))
#4	#3 OR #2 OR #1
#3	TS=("motor complete")
#2	TS=(parapleg* or quadripares* or quadriparet* or quadripleg* or tetrapares* or tetraparet* or tetraplag* or tetrapleg* or quadri-pares* or quadri-paret* or quadri-pleg* or tetra-pares* or tetra-paret* or tetra-plag* or tetra-pleg*)
#1	TS=(paralys* or paralyt* or paralyz*)
